# The Impact of Dual‐Salt Electrolyte with Low Fluorine Content on the Performance of Layered Transition Metal Oxides for Sodium‐Ion Batteries

**DOI:** 10.1002/smll.202410704

**Published:** 2025-05-08

**Authors:** Yiyue Lu, Muhammad Nouman Aslam, Christian Leibing, Maider Zarrabeitia, Ludovica Roselli, Lukas Fridolin Pfeiffer, Peter Axmann, Jonas Geisler, Philipp Adelhelm, Andrea Balducci

**Affiliations:** ^1^ Institute of Technical and Environmental Chemistry Friedrich Schiller University Jena and Center for Energy and Environmental Chemistry (CEEC) Jena Philosophenweg 7a 07743 Jena Germany; ^2^ Helmholtz Institute Ulm (HIU) Helmholtzstrasse 11 89081 Ulm Germany; ^3^ Karlsruhe Institute of Technology (KIT) P.O. Box 3640 76021 Karlsruhe Germany; ^4^ ZSW Center for Solar Energy and Hydrogen Research Baden‐Württemberg Helmholtzstraße 8 80801 Ulm Germany; ^5^ Institut für Chemie Humboldt‐Universität zu Berlin Brook‐Taylor‐Str. 2 12489 Berlin Germany; ^6^ Joint research group Operando Battery Analysis (CE‐GOBA) Helmholtz‐Zentrum Berlin Hahn‐Meitner‐Platz 1 14109 Berlin Germany

**Keywords:** electrolyte, propylene carbonate, sodium bis(fluorosulfonyl)imide, sodium difluoro(oxalato)borate, sodium‐ion battery

## Abstract

In this work, the characterization of novel electrolytes based on the combination of propylene carbonate (PC) solvent with sodium bis(fluorosulfonyl)imide (NaFSI) and sodium difluoro(oxalato)borate (NaDFOB), as well as their application in sodium‐ion batteries (SIBs) is presented. The results show that dual‐salt electrolytes have a wide electrochemical stability window, excellent transport properties, and mostly suppress anodic dissolution. When combined with P2‐Na_2/3_Al_1/9_Fe_1/9_Mn_2/3_Ni_1/9_O_2 _(P2‐AFMNO) cathode electrode for SIBs operating at 4.3 V vs Na^+^/Na, they enable high performance and stability. XPS investigation revealed that this performance is related to the formation of a thin and homogeneous cathode electrolyte interphase (CEI) at the electrode surface.

## Graphical abstract

Dual‐salt electrolytes with low fluorine content high voltage sodium‐ion batteries cathodes: In this work, novel electrolytes containing two low fluorine content salts with enhanced safety features and the ability to passivate the Al current collector is reported. The suitability of these electrolytes in combination with P2‐Na_2/3_Al_1/9_Fe_1/9_Mn_2/3_Ni_1/9_O_2 _(P2‐AFMNO) cathode operating at 4.3 V is demonstrated and outperforms reference electrolyte formulations due thin and homogeneous cathode electrolyte interphase (CEI) at the electrode surface.

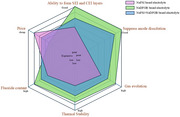

## 1 Introduction

Sodium‐ion batteries (SIBs) are currently considered the most viable alternative to lithium‐ion batteries (LIBs).^[^ ^1^ ^]^ Although the theoretical energy density of SIBs is lower than that of LIBs based on Lithium nickel manganese cobalt oxides cathode,^[^ ^2^ ^]^ they are competitive with LiFePO_4_‐based LIBs. Most importantly, SIBs offer an opportunity to alleviate the problems associated with the supply, cost and toxicity of raw elements and electrode materials used in LIBs.^[^ ^3^ ^]^ For these reasons, significant efforts have been devoted to the development of high‐performance SIBs over the past decade, culminating in the beginning of the commercialization of these devices.

SIBs typically contain hard carbon as anode material,^[^ ^4^ ^]^ and a Prussian blue derivative,^[^ ^5^ ^]^ a polyanionic compound^[^ ^6^ ^]^ or a layered transition metal oxide^[^ ^7^ ^]^ as the cathode active material. As the electrolyte, sodium hexafluorophosphate (NaPF_6_) dissolved in mixtures of ethylene carbonate (EC) and propylene carbonate (PC) is widely used, which may be considered analogous to LP30 (1 M LiPF_6_ in EC: DMC) used in LIBs. In LIBs, the composition of the electrolyte has a significant impact on the electrochemical performance as well as on the safety of the battery. In sodium technology this is even more crucial due to the differences in the chemical and stability properties of the degradation products of the electrolyte, such as the formation of the inorganic‐rich layer, mainly composed of highly soluble Na_2_CO_3_. Although state‐of‐the‐art electrolyte formulations based on organic carbonate solvents can guarantee good performance, they still have limitations in terms of safety and stability.^[^ ^8^ ^]^ In fact, although fluorine is favorable for the formation of stable SEI,^[^ ^9^ ^]^ the high fluorine content of NaPF_6_ presents significant environmental and safety concerns. For this reason, the development of novel electrolytes having a limited fluorine content (with respect to existing formulations) appears of great importance for the future of safe and sustainable SIBs.

**Figure 1:**
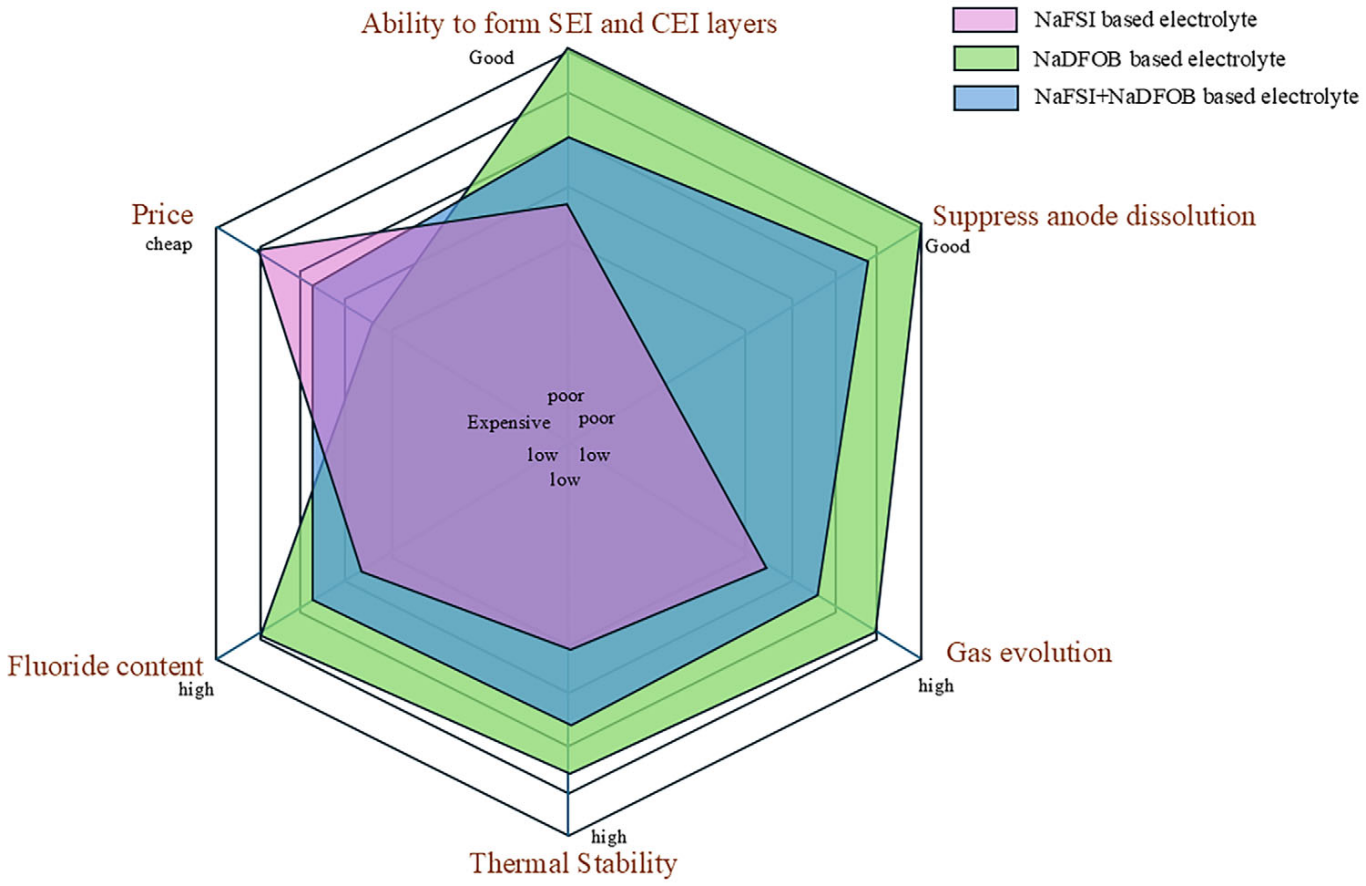
Synergistic effect of NaFSI and NaDFOB in the development of low‐fluorine‐content electrolytes.

Among the various salts investigated so far, sodium bis(fluorosulfonyl)imide (NaFSI) and sodium difluoro(oxalato)borate (NaDFOB) appear of particular interest. Both have a limited fluorine content and are not considered as per‐ and polyfluoroalkyl substances. The price of NaFSI (5.8 €/g)^[^ ^10^ ^]^ is rather low (comparable to PF_6_‐based salt, which is 8.24 €/g^[^ ^11^ ^]^) and this salt exhibits the typical favorable properties of imide‐based salts, including thermal stability and high solubility.^[^ ^12^ ^]^ However, its use at high voltages is problematic due to its limited ability to prevent the anodic dissolution of Al current collectors, a limitation common to imide salts.^[^ ^13^ ^]^ In contrast, NaDFOB can form a good solid electrolyte interphase (SEI) and has the ability to protect the Al current collector.^[^ ^14^, ^15^, ^16^, ^17^, ^18^ ^]^ However, its use might promote unwanted gas evolution within the cell.^[^ ^19^ ^]^ Taking these properties into account, as illustrated in **Figure** **1**, it is evident that the combined use of these salts (i.e., NaFSI and NaDFOB) could be promising for the development of innovative low‐fluorine content electrolytes. In the past, Ciucci's group considered the combination of NaTFSI and NaDFOB in polymer electrolytes.^[^ ^20^ ^]^ Recently, Müller‐Buschbaum and Xia's research groups reported interesting results using the electrolyte 0.8 m NaFSI + 0.2 m NaDFOB (EC:PC (1:1, v/v)) in combination with Na₃V₂(PO₄)₃ (polyanionic compound) cycled between 2.2 and 4.2 V vs Na⁺/Na. They focused on explaining the reaction mechanism and the reasons why NaDFOB forms stable CEI and SEI layers.^[^ ^21^ ^]^ However, additional investigations are needed to understand the advantages and limits associated with the simultaneous use of these two salts, especially when used in combination with P2‐NaAFMNO (layered transition metal oxide) cathode materials operating above 4.0 V, as these materials are particularly promising for the realization of high energy SIBs.^[^ ^22^, ^23^ ^]^

Herein, we report an investigation on the use of three electrolytes, namely 1 m NaFSI in PC, 0.8 m NaFSI + 0.2 m NaDFOB in PC and 0.5 m NaFSI + 0.5 m NaDFOB in PC, in combination with a layered oxide‐based cathode material (P2‐Na_2/3_Al_1/9_Fe_1/9_Mn_2/3_Ni_1/9_O_2_). The aim of the proposed formulations was to understand the influence of the addition of NaDFOB on the electrochemical and interfacial properties of FSI‐based electrolytes for SIBs. With this aim, we initially investigated the transport and thermal properties of the three electrolytes, as well as their ability to prevent the anodic dissolution of Al. Afterward, their impact on the initial Coulombic efficiency, capacity, capacity retention, and cycling stability of the cathode has been considered in detail. Finally, we extensively studied the influence of the electrolyte formulation on the cathode electrolyte interphase (CEI) and preliminary studies on the gas evolution occurring at the cathode have been conducted.

## 2 Results and Discussion

### 2.1 Chemical‐Physical Characterization of the Electrolytes

**Figure 2:**
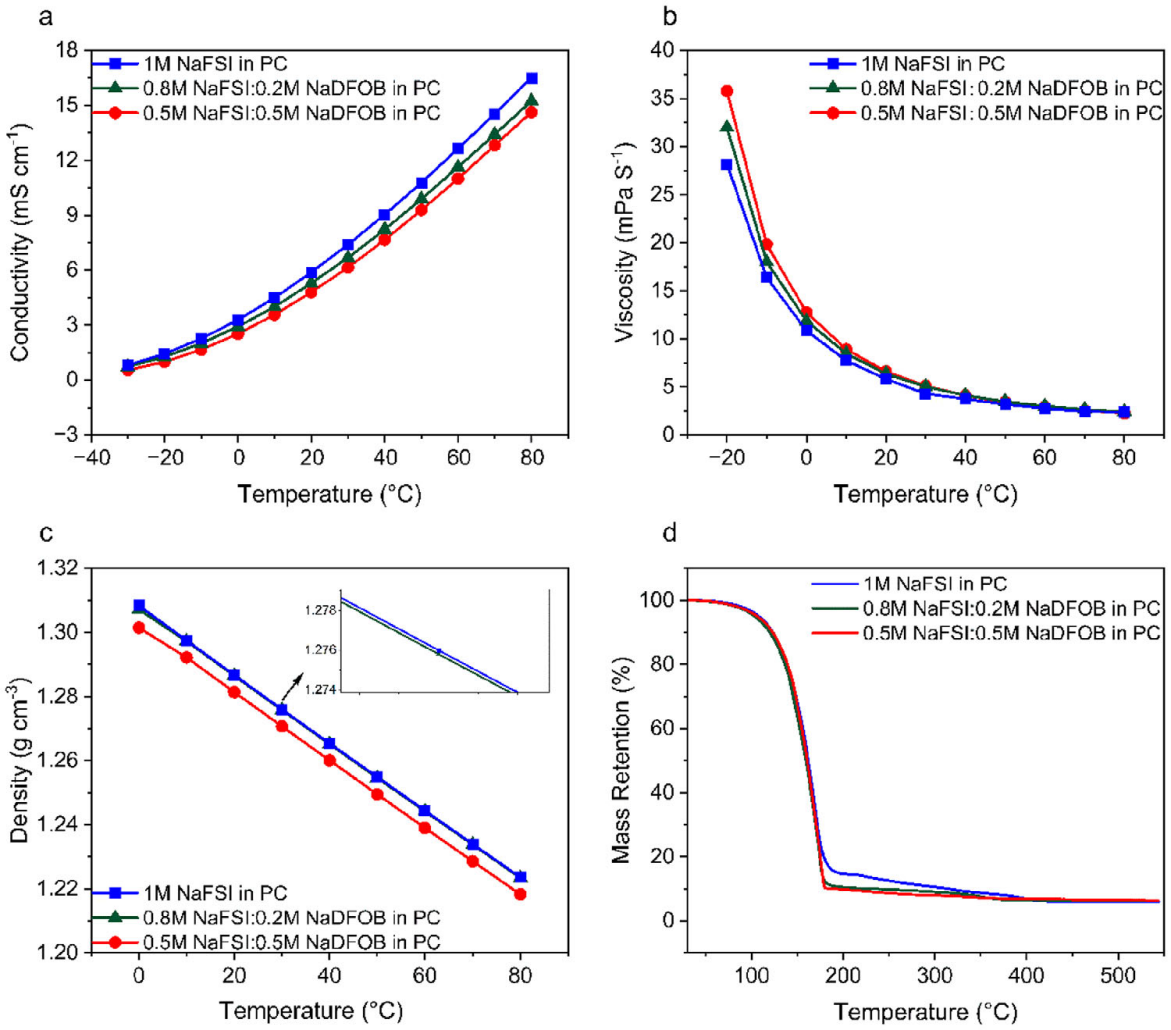
a) Conductivity of the investigated electrolytes between −30 and 80 °C; b) viscosity of the investigated electrolytes between −20 and 80 °C; c) density of the investigated electrolytes between 0 and 80 °C; d) dynamic TGA curve in a temperature range from 30 to 550 °C in nitrogen atmosphere.

**Figure** **2** compares the temperature dependency of conductivity, viscosity, density and thermal stability of the three investigated electrolytes. As shown in Figure 2a, the addition of NaDFOB to 1 m NaFSI in PC slightly reduces the conductivity, but overall, the three investigated electrolytes display a very similar conductivity throughout the investigated temperature range (−30 to 80 °C). At 20 °C, the conductivity of 1 m NaFSI in PC, 0.8 m NaFSI + 0.2 m NaDFOB in PC, and 0.5 m NaFSI + 0.5 M NaDFOB in PC was 5.9, 5.3, and 4.8 mS cm^−1^, respectively. These values are comparable to those of other PC‐based electrolytes reported in the literature.^[^ ^24^ ^]^ The viscosity of the electrolytes is also very similar. Still, in this case, the addition of NaDFOB to the electrolytic solution slightly increases the viscosity values (see Figure 2b). At 20 °C, 1 m NaFSI in PC, 0.8 m NaFSI + 0.2 m NaDFOB in PC, and 0.5 m NaFSI + 0.5 m NaDFOB in PC exhibit a viscosity of 5.8, 6.4, and 6.6 mPa s^−1^, respectively. This behavior is caused by the fact that the Na⁺ cation exhibits strong interactions with solvent molecules due to its high charge density. The DFOB⁻ anion, which contains both oxalate and difluoro groups, also has strong coordinating ability. These interactions reduce the free movement of solvent molecules, effectively increasing the solution's viscosity and slowing ion migration. As a result, the addition of NaDFOB increases the electrolyte's viscosity while simultaneously decreasing its ionic conductivity. The presence of NaDFOB in the electrolyte decreases also the density of the solution, and at 20 °C, the three electrolytes display a density comprised between 1.28 and 1.29 g cm^−3^_,_ as can be seen in Figure 2c. Figure 2d compares the thermal stability of the investigated electrolytic solutions. The mass retention of 1 m NaFSI in PC levels off at 190 °C, whereas the electrolytes containing NaDFOB started to lose weight at 180 °C. Interestingly, between 300 and 400 °C, the mass retention of all three electrolytic solutions decreased to varying extents. This different behavior is caused by the different decomposition of the salt and the fact that NaDFOB is decomposing at a slightly lower temperature than NaFSI (see Figure S1 of Supporting Information).^[^ ^25^ ^]^ The isothermal measurements at 60 °C (see Figure S2 in Supporting Information) confirm that the thermal stability of all electrolytes is well above the operating standards of SIBs.

**Figure 3:**
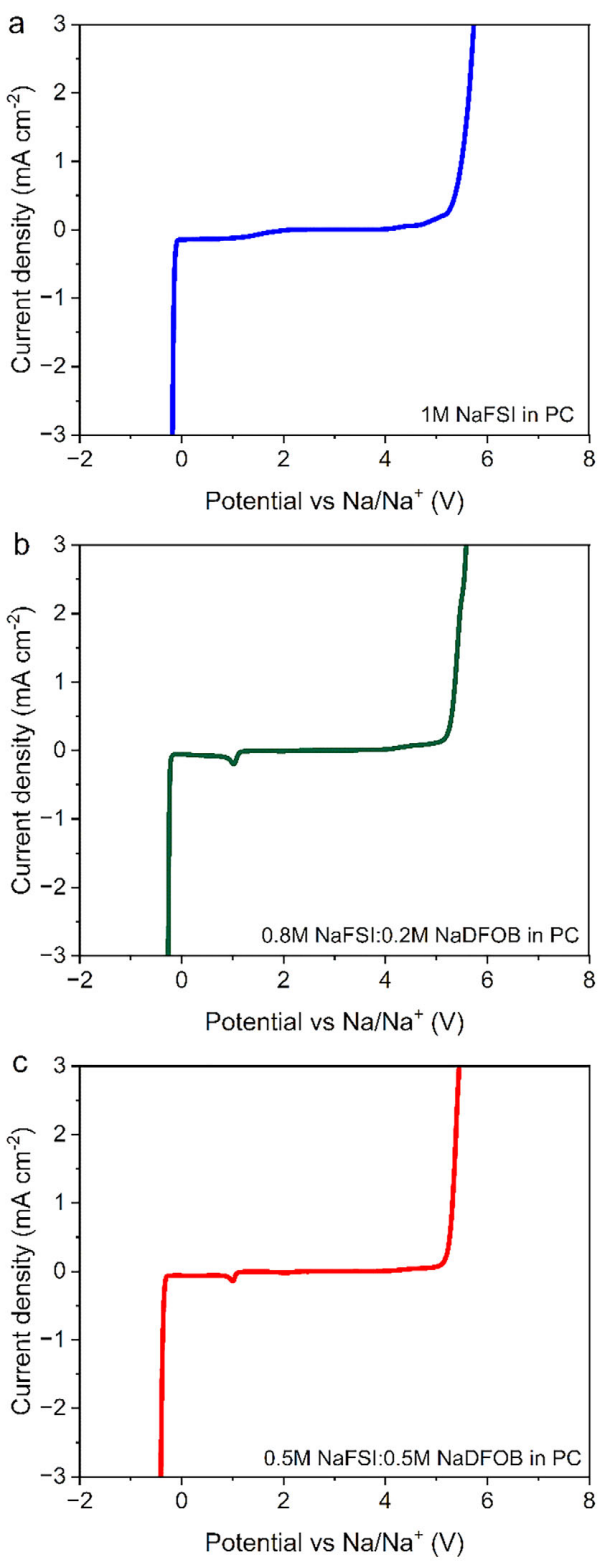
Overall electrochemical stability of a) 1 m NaFSI in PC; b) 0.8 m NaFSI + 0.2 m NaDFOB in PC; and c) 0.5 m NaFSI + 0.5 m NaDFOB in PC.

As shown in **Figure** **3**, the overall electrochemical stability of all electrolytes is ≈5 V vs Na^+^/Na. It is important to observe, however, that the 1 m NaFSI in PC electrolyte displays an increase in current at 4.2 V vs Na^+^/Na. This increase, which was already observed by Passerini's group,^[^ ^26^ ^]^ indicated that the FSI anion becomes electrochemically active at this potential. This instability might cause problems when this electrolyte is used in combination with a high‐voltage cathode. The addition of NaDFOB into the electrolytic solution leads to two concomitant effects. On one hand, the anodic stability of the electrolytes increases, delivering higher oxidation stability, and suggesting it may be a better choice for high‐voltage cathodes. This behavior was also reported for lithium‐based electrolytes.^[^ ^27^ ^]^ On the other hand, the appearance of a peak at ca. 1.0 V vs Na^+^/Na could be associated with the decomposition of the DFOB anion and the formation of a passivation layer. This behavior has already been reported for lithium‐based systems.^[^ ^28^, ^29^ ^]^ Taken together, these results suggest that the addition of NaDFOB appears to improve the overall stability of the electrolyte, especially in terms of anodic stability.

### 2.2 Anodic Dissolution of Al Current Collectors

To gain further insight into the influence of NaDFOB on the electrochemical stability, the ability of the three electrolytes to suppress anodic dissolution of Al current collectors was investigated. The process was investigated by applying a constant potential of 4.3 V (vs Na^+^/Na) to uncoated aluminum disks and measuring the resulting change in current at a scan rate of 0.5 mV s^−1^,^[^ ^30^ ^]^ in order to minimize the influence of polarization resistance on the anodic limit of the electrolyte.^[^ ^31^ ^]^

**Figure 4:**
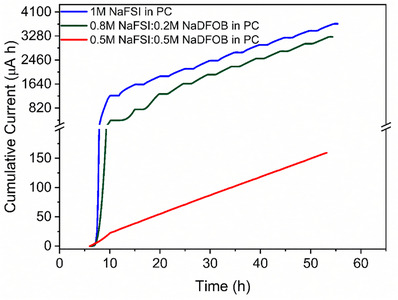
Current evolution response from the anodic dissolution tests performed with the different electrolyte systems.

As shown in **Figure** **4** (more details are available in Figures S3 and S4 and S5 of Supporting Information), the 1 m NaFSI in PC electrolyte shows a pronounced anodic current at 4.3 V, indicating the occurrence of Al dissolution. This behavior is aligns with previously reported results in the literature.^[^ ^14^, ^15^, ^16^, ^17^, ^29^, ^32^ ^]^ The addition of NaDFOB to the electrolyte results in a reduction in the recorded anodic current. Herein, the correlation between salt concentration and magnitude of current is inverse. Consequently, increasing concentrations of NaDFOB can minimize the magnitude of the anodic dissolution process, although the process cannot be completely suppressed.

**Figure 5:**
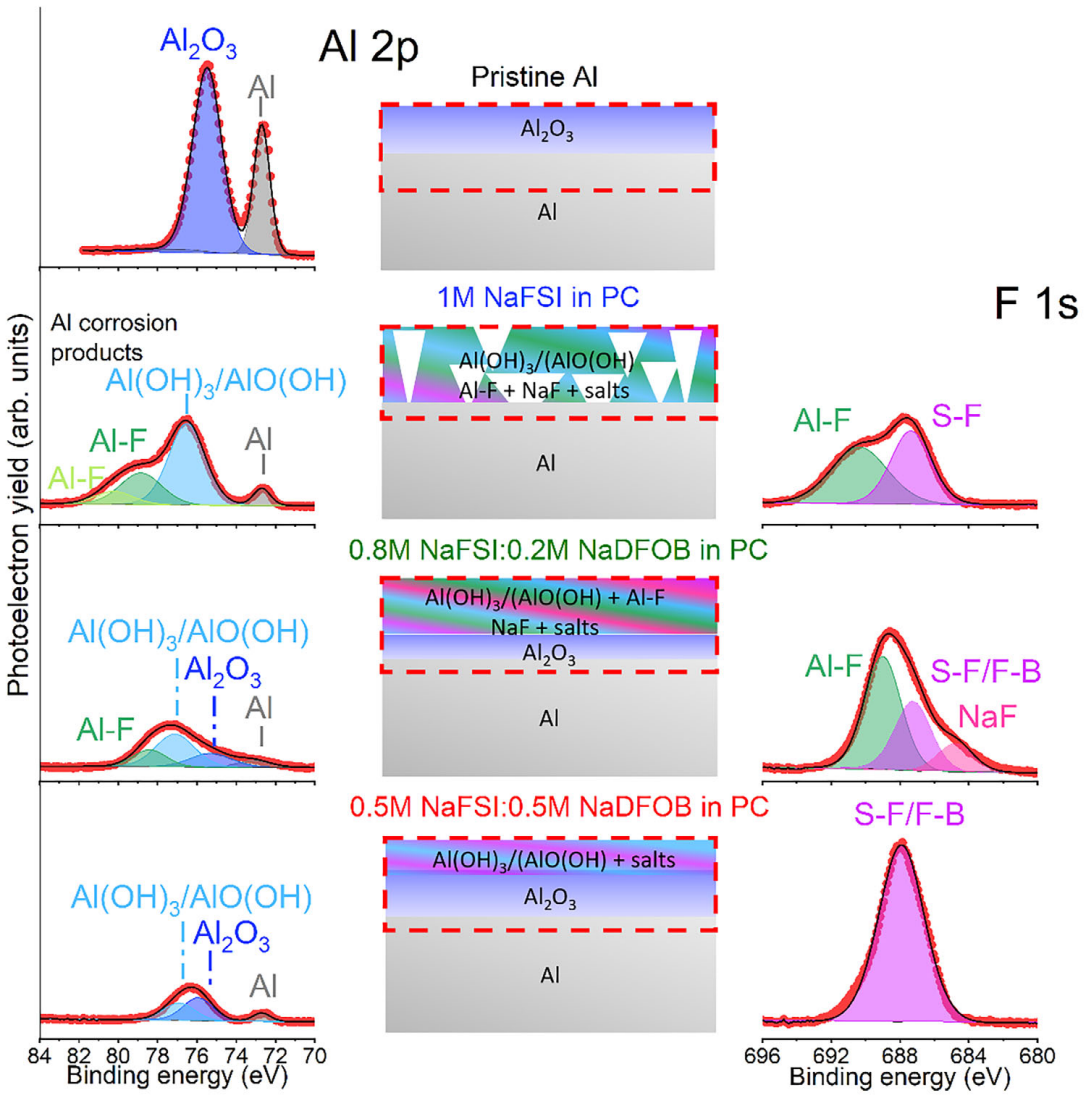
XPS spectra of Al 2p and F 1s photoelectron regions of the pristine and cycled Al current collectors in different electrolytes, as well as the scheme of the formed interface.

The Al corrosion has also been investigated by analyzing the surface chemistry of ex situ cycled Al current collectors in three electrolyte formulations by ex situ XPS (**Figure** **5**). The cells were cycled over 10 cycles at 0.5 mV s^−1^ from 2.3 to 4.3 V, holding the potential for 3 h at 4.3 V in each cycle. The Al 2p and F 1s regions of pristine and cycled Al current collectors are illustrated in Figure 5. The pristine Al current collector exhibits characteristic Al metal and Al_2_O_3_ peaks.^[^ ^33^ ^]^ In contrast, the cycled Al current collectors display low‐intensity Al metal and Al_2_O_3_ signals due to the Al corrosion, forming hydroxides, such as (Al(OH)_3_ and AlO(OH) due to the reactions with water traces in the electrolyte.^[^ ^34^ ^]^ Moreover, the Al current collector tested in 1 m NaFSI in PC and 0.8 m NaFSI + 0.2 m NaDFOB in PC shows aluminum fluoride (Al‐F) species from the decomposition of NaFSI and/or NaDFOB salts.^[^ ^35^ ^]^ The Al current collector tested in 1 m NaFSI in PC shows a higher concentration of hydroxide and fluoride species than the Al current collectors tested in NaDFOB‐containing electrolytes (17.6% vs 9.0% and 3.3%, respectively). Indeed, the Al current collector tested in 1 m NaFSI in PC does not display the Al_2_O_3_ peaks, suggesting the full transformation of oxide to hydroxide and fluorine‐based species due to Al corrosion. However, it also shows higher Al metal concentration than the Al ones tested in the presence of NaDFOB, suggesting that the formed interphase, in this case, is rather inhomogeneous, destroying the Al_2_O_3_ passivation surface and not preventing the Al corrosion. The Al current collector tested in NaDFOB‐based electrolytes revealed that the Al corrosion formed layer is more homogeneous, as well as increasing the NaDFOB concentration leads to a significant reduction in Al corrosion, in agreement with anodic dissolution tests.

The results discussed above show that the three investigated electrolytes display promising transport and thermal properties. Although the presence of NaDFOB reduces the conductivity and increases the viscosity, its addition to the electrolyte improves the electrochemical stability and suppresses the anodic dissolution of Al, as also confirmed by the XPS results.

### 2.3 Electrochemical Performance of P2‐AFMNO Cathodes

**Figure 6:**
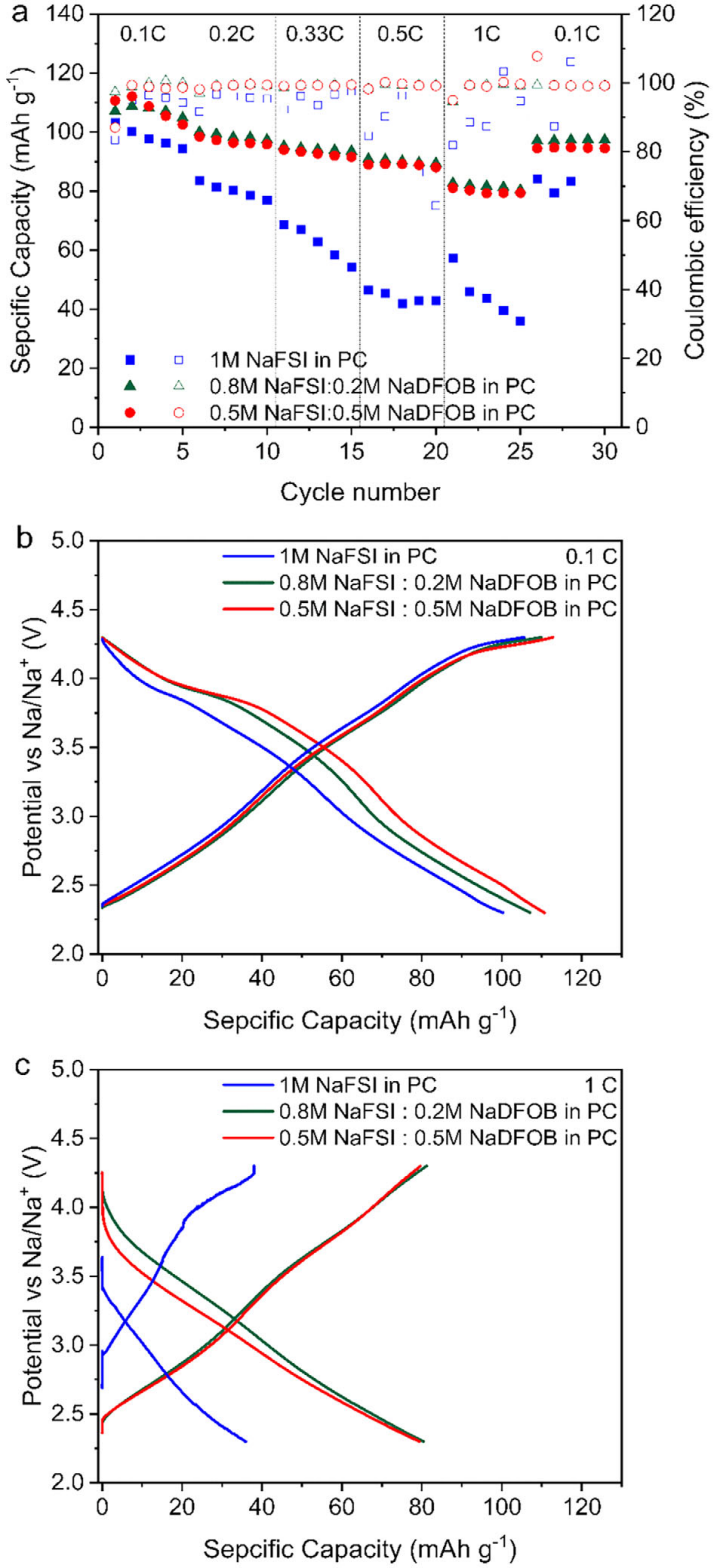
Comparison of a) capacity retention and galvanostatic charge/discharge voltage profile at 0.1C (b) and 1C (c) of P2‐AFMNO electrode in the investigated electrolytes.

**Figure** **6**a shows a comparison of the capacity retention displayed by the P2‐AFMNO electrodes in the three electrolytes during charge‐discharge tests carried out at different C‐rates (more details are available in Figure S6 of Supporting Information). The P2‐AFMNO electrodes were cycled between 2.3 and 4.3 V vs Na/Na^+^. As shown, during the initial cycles at 0.1 C the P2‐AFMNO electrode cycled in the 1 m NaFSI in PC displays a capacity of ca. 100 mAh g^−1^. The P2‐AFMNO electrodes cycled in the electrolytes containing NaDFOB display higher capacity, in the order of 107 and 110 mAh g^−1^ for 0.2 m or 0.5 m NaDFOB containing electrolytes, respectively. When the C‐rate increases, the electrochemical response of the P2‐AFMNO electrodes is strongly affected by the electrolyte composition. In fact, the P2‐AFMNO electrode cycled on 1 m NaFSI in PC displays a rather low‐capacity retention, and at 1 C it delivers only 40 mAh g^−1^, losing 56% respect to the initial capacity. On the other hand, the P2‐AFMNO electrodes cycled on the NaDFOB containing electrolytes displayed much higher capacity retention (ca. 78%), and at 1 C they deliver a capacity of 80 mAh g^−1^. These higher values are certainly promising, indicating the better rate capability of the P2‐AFMNO cathode electrodes by modifying the electrolyte. Figure 6b,c shows a comparison of the voltage profile of the electrode at different currents, *i.e*., 0.1 C and 1 C, respectively. As shown, the addition of NaDFOB into the electrolytic solution leads to superior cycling performance and improved higher reversibility during charge‐discharge tests at 1 C. It is worth noting that a plateau at high voltage was observed for P2‐AFMNO in all three electrolytes during charge and discharge at low C‐rates (*e.g*. 0.1 C). In these cycles, the obtained charge and discharge capacity in the three electrolytes differs mainly by the capacity in the high‐voltage region (> 3.8 V vs Na^+^/Na). When the C‐rate is increased (*e.g*., 1 C in Figure 6c), a significant polarization is observed when 1 m NaFSI in PC is used as electrolyte. With increasing concentration of NaDFOB the polarization decreases. Both observations suggest the formation of a more effective CEI when NaDFOB is added to the electrolyte.

**Figure 7:**
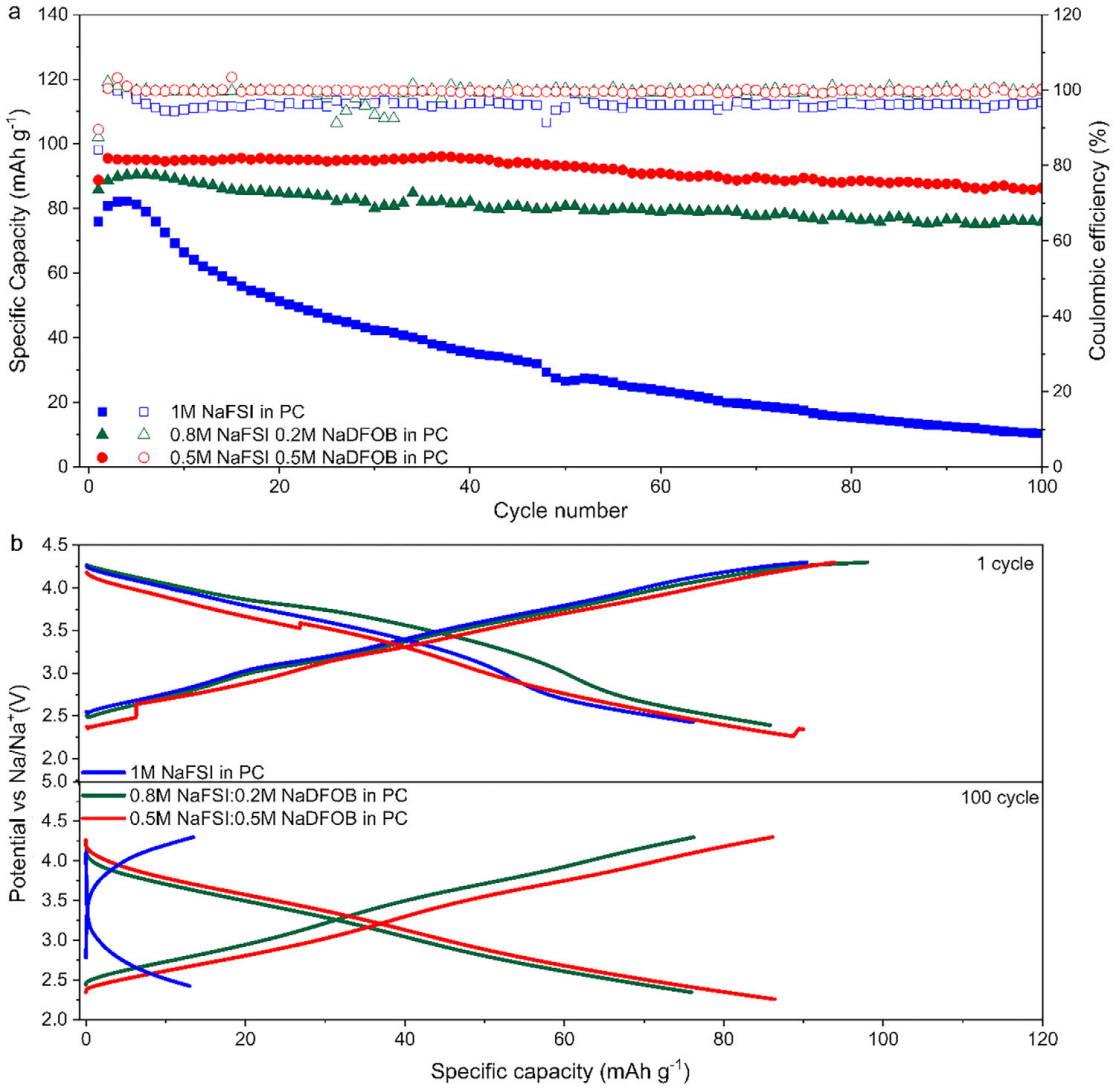
a) Capacity retention of P2‐AFMNO electrodes in the investigated electrolytes during charge–discharge test carried out at 0.5 C; b) Evolution over the cycling of the charge‐discharge profiles.

These results indicate that although 1 m NaFSI in PC displays the most favorable transport properties, its use is limited by its instability at high voltage. This instability is also strongly affecting the cycling stability of the P2‐type cathode materials. As shown in **Figure** **7**a, the P2‐AFMNO electrode cycled in 1 m NaFSI in PC loses nearly all its initial capacity after 100 cycles at 0.5 C. The P2‐AFMNO electrodes cycled in the electrolytes containing NaDFOB, on the contrary, exhibit excellent capacity retention (86% and 91% for 0.2 and 0.5 m containing NaDFOB electrolytes, respectively). Among them, the P2‐AFMNO electrode cycled in 0.5 m NaFSI + 0.5 m NaDFOB in PC displays the highest initial capacity (95 mAh g^−1^ at 0.5 C) and the best capacity retention (>90% after 100 cycles). To the best of our knowledge, this performance is among the highest reported for this type of cathodic material when cycled up to 4.3 V vs Na^+^/Na.^[^ ^23^, ^36^, ^37^ ^]^ For instance, Zhang's group employed P2‐NLMNC as the cathode material and 1 m NaClO₄ in EC: PC (1:1 vol%) with 5 vol% FEC as the electrolyte. After 100 cycles, the NLMNC demonstrated a capacity retention of 82.5%, indicating good cycling stability.^[^ ^23^ ^]^ Figure 7b compares the charge‐discharge profiles of the investigated electrodes at the beginning and the end of the cycling process. As shown, the electrodes cycled in the electrolytes containing NaDFOB maintain their potential profiles over course of 100 cycles. The electrode cycled in 1 m NaFSI in PC, on the other hand, displays a significant decrease in the reversible capacity. The different behavior of these electrodes is well visible in Figure S8a,b (Supporting Information), which compare the evolution of the differential capacity of the P2‐AFMNO electrodes during the cycling process (1st vs 100th cycle). In 1 m NaFSI in PC the position and the delivered differential capacity of the redox/phase transition/s peaks shifted and decreased considerably after 100 cycles, indicating that in this electrolyte, the P2‐AFMNO material underwent a phase change and significant polarization. Therefore, the addition of NaDFOB promotes the formation of a stable CEI layer, reducing continuous electrolyte decomposition and unwanted side reactions at the electrode surface, thereby enhancing cycle life and Coulombic efficiency. The participation of DFOB⁻ in surface passivation minimizes metal dissolution and inhibits dendrite growth. Moreover, the presence of DFOB⁻ improves the oxidative stability of the electrolyte, enabling superior performance at high voltages.

### 2.4 Chemical Composition of the Cathode Electrolyte Interphase of Cycled P2‐AFMNO Electrodes

**Figure 8:**
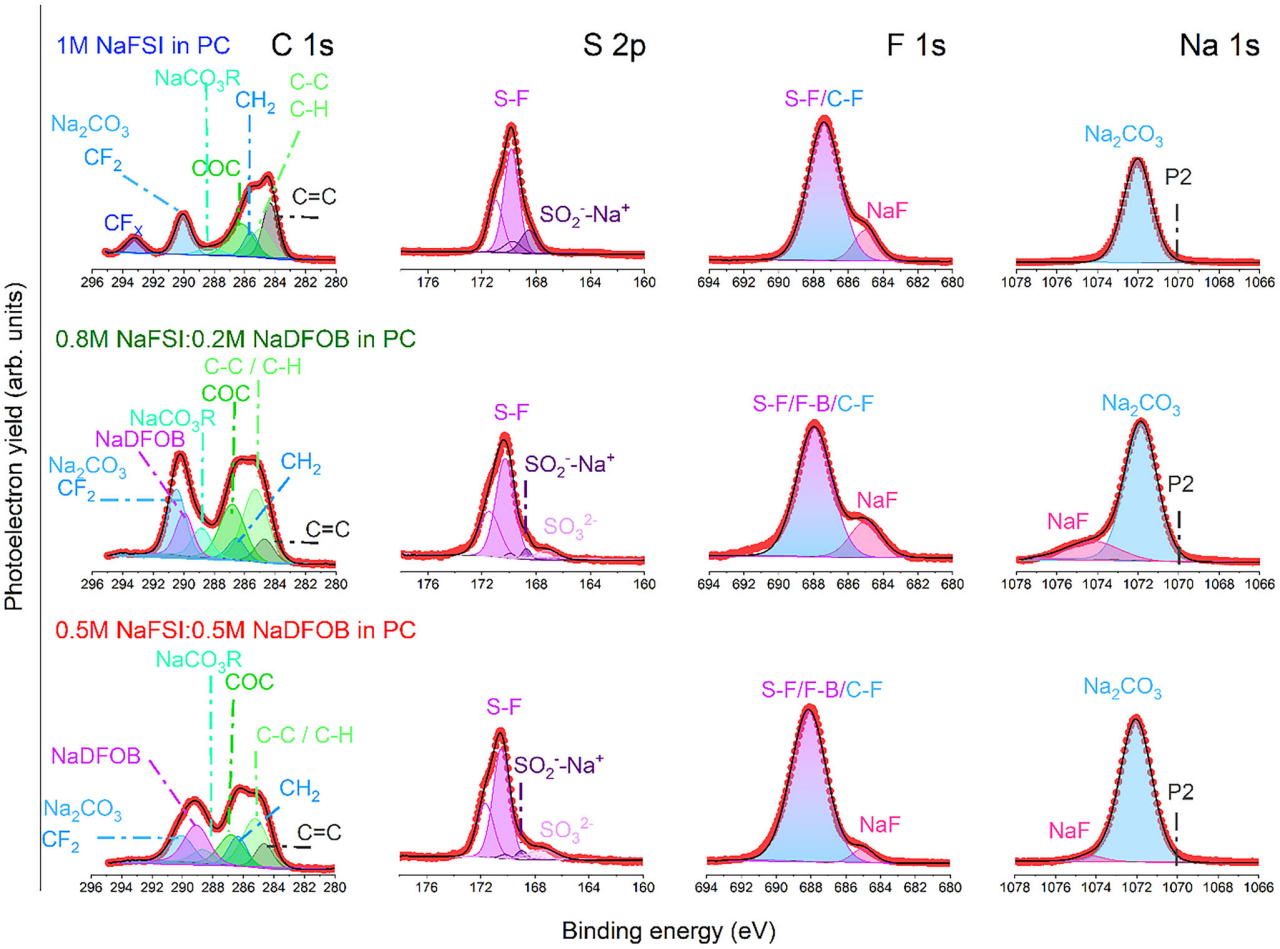
XPS spectra of C 1s, S 2p, F 1s, and Na 1s photoelectron regions of the cycled P2‐AFMNO cathode electrode in the three investigated electrolytes.

The chemical composition of the formed CEI onto cycled P2‐AFMNO electrodes in the three investigated electrolytes was analyzed by XPS. The comparison of C 1s, F 1s, S 2p, and Na 1s spectra after cycling is presented in **Figure** **8**. The C 1s spectra show similar CEI species, such as C═C (284.4 eV), C‐C/C‐H (285.0 eV), CH_2_ (286 eV), COC (286.5), NaCO_3_R (288.5 eV), Na_2_CO_3_ and CF_2_ (290 eV) corresponding to the conductive carbon,^[^ ^38^, ^39^ ^]^ PVDF binder, and reduced species from the PC solvent. However, they differ in the concentration of these species on the CEI. Indeed, the P2‐AFMNO electrodes tested in NaDFOB‐containing electrolytes display a higher concentration of species appearing at high binding energies (>288 eV), such as Na_2_CO_3_ and NaDFOB, suggesting a richer inorganic CEI, in agreement with the previous results.^[^ ^17^ ^]^ In addition, the C 1s of P2‐AFMNO cycled in 1 m NaFSI in PC reveals the presence of carbon‐fluorine species, which are formed due to the decomposition of the PVDF binder, indicating poor stability of the electrode in this electrolyte. This might be one of the reasons for the poor capacity retention observed for P2‐AFNMO in 1 m NaFSI in PC. The S 2p region has also been investigated, showing similar sulfur‐based species on the CEI, mainly S‐F and SO_2_^−^Na^+^ species from NaFSI and its decomposition product, respectively.^[^ ^40^ ^]^ In addition, the CEI formed onto P2‐AFMNO tested in NaDFOB‐containing electrolytes exhibit an extra peak at lower binding energy, i.e., 167.4 eV, which might be related to sulfite (SO_3_^2−^), suggesting once more the more inorganic‐rich CEI.

The F 1s region shows the presence of S‐F, B‐F, and/or C‐F bonds from NaFSI, NaDFOB, and PVDF, as well as NaF (at 684 eV) decomposition product.^[^ ^41^ ^]^ The presence of NaF on the CEI corresponds to the PVDF dehydrofluorination and NaFSI reduction.^[^ ^42^ ^]^However, the degradation of NaDFOB could not be discarded, as suggested by the absence of a B‐F bond on the surface of the P2‐AFMNO electrode tested in 0.8 m NaFSI + 0.2 m NaDFOB in PC (see Figure S10, Supporting Information), also not observed in similar electrolyte composition.^[^ ^43^ ^]^ The main difference is the lower NaF concentration observed in the P2‐AFMNO tested in 0.5 m NaFSI + 0.5 m NaDFOB in PC electrolyte, which might be due to the thicker (the NaF is usually found closer to the electrode) or more homogeneous CEI (note that homogeneous outermost layer could cover the inner NaF layer being less detectable by XPS). The Na 1s is a very surface‐sensitive region, providing the chemistry of the outermost layer. Considering the Na 1s spectrum of P2‐AFMNO in the mentioned electrolyte shows the presence of NaF, it might be concluded that the latter hypothesis is more plausible, suggesting that the CEI is rather thin but homogeneous, in agreement with the better electrochemical performance observed for P2‐AFMNO in 0.5 m NaFSI + 0.5 m NaDFOB in PC. Please note that the Na 1s photoelectron is a surface‐sensitive region. Therefore, the absence of the NaF XPS peak in Na 2s sectra for the 1 m NaFSI in PC system indicates the formation of a thicker layer (NaF formation is usually observed at higher depths, close to the electrode). Another possible reason might be related to the formation of insoluble BOB‐Mn(II) species, as reported before,^[^ ^17^ ^]^ which stabilize the CEI. The surface of P2‐AFMNO tested in 0.5 m NaFSI + 0.5 m NaDFOB in PC electrolyte displays Mn 2p signal; however, the O 1s region indicates the absence of exposed P2‐AFMNO, in contrast with the P2‐AFMNO tested in 1 m NaFSI in PC, which exhibits the P2 signal, as well as lower Mn content (Figure S11, Supporting Information). This suggests the formation of a new Mn‐based species in the formed CEI in 0.5 m NaFSI + 0.5 m NaDFOB in PC electrolyte, which likely corresponds to the formation of insoluble BOB‐Mn(II) species, also leading to a better electrochemical performance. Regarding the thickness of the CEI, the three systems show a rather thin CEI. In fact, although the XPS cannot provide accurate values, it can be estimated from the photoelectron escape length, considering the inelastic mean free path (IMFP) and the photon energy of specific chemical bonds.^[^ ^44^ ^]^ Hence, considering the used photon energy and the IMFP of the C═C bond (95% of the photoelectrons detected originate within 7 nm), it can be concluded that the formed three CEIs are thinner than 7 nm.

### 2.5 Differential Electrochemical Mass Spectrometry

Differential electrochemical mass spectrometry (DEMS) studies were performed to provide information on the gas release and composition that occurs when the stability limit of the electrolytes is exceeded. DEMS is a powerful tool to study the onset of electrolyte decomposition and the data can be quantitative, providing insight not only into the stability limit but also into the decomposition reactions.

**Figure 9:**
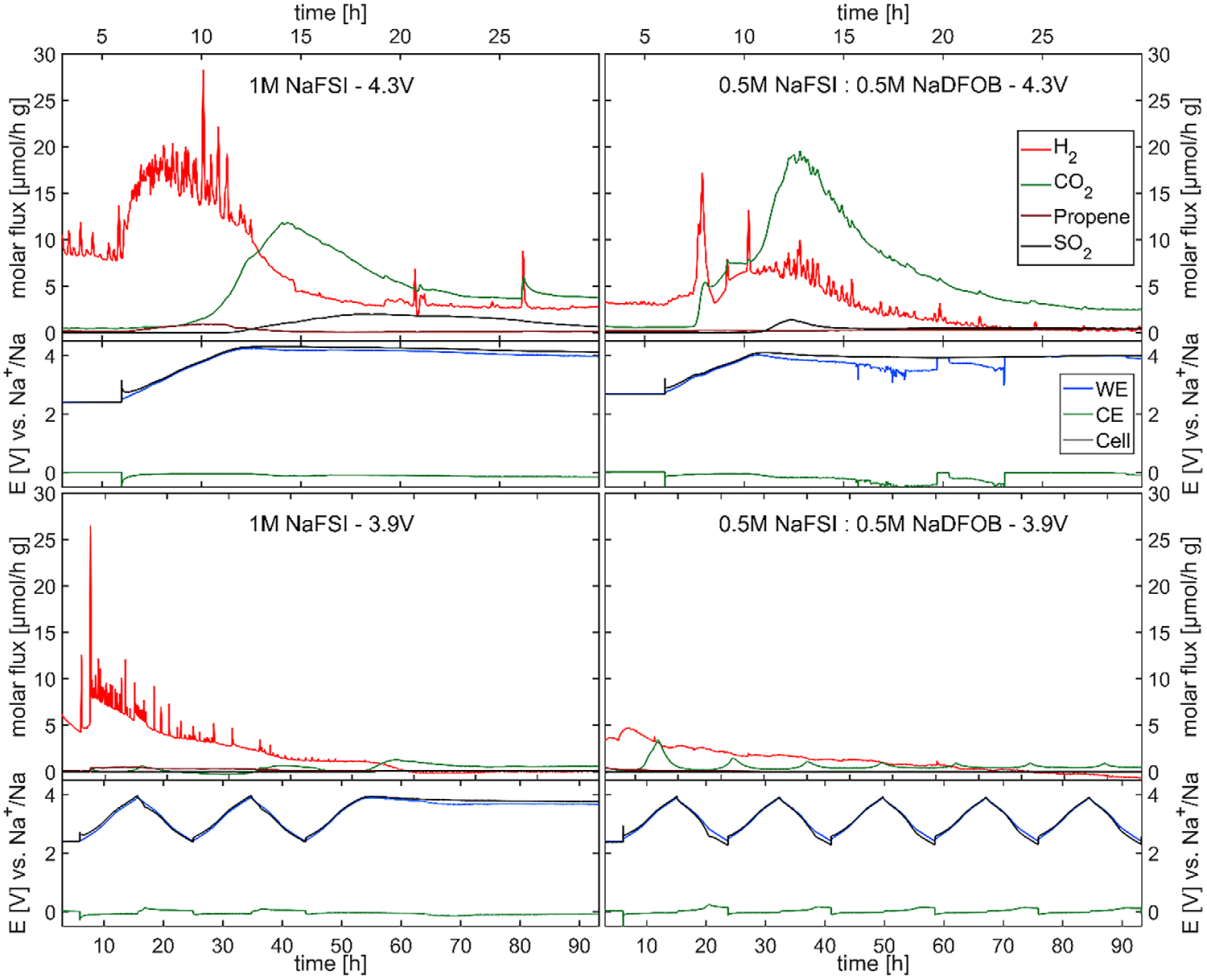
Operando‐DEMS measurements during galvanostatic cycling for different electrolytes, 1 m NaFSI and 0.5 m NaFSI + 0.5M NaDFOB in PC, for different upper cut‐off potentials 4.3 and 3.9 V respectively. Measured in three‐electrode configuration. Potentials given for P2‐AFMNO working electrodes (WE) and sodium metal counter electrode (CE) against sodium metal reference and the resulting cell potential (Cell). Although the total amounts of gas evolution are given for guidance, some measurements are only pseudo‐quantitative, detailed information in the supporting information.

As visible in **Figure** **9**, the comparison of the different electrolytes shows more initial CO_2_ evolution for the 0.5 m NaFSI + 0.5 m NaDFOB electrolyte in both measurements than for their respective measurements with pure NaFSI in PC. While the initial CO_2_ formation is higher, a faster decay can be observed for the NaDFOB containing electrolyte. This could be an indication that the associated reaction in the case of the mixed electrolyte leads to a stronger CEI formation and ultimately a better protected electrode. This is consistent with the observation from the XPS measurements of a carbonate‐rich CEI in the case of the mixed electrolyte. In the case of the pure NaFSI small amounts of propene are found which are not observed in the mixed electrolyte and the formation of H_2_ is higher. This also indicates a different path of PC degradation in the absence of NaDFOB.

While differences in the electrolyte decomposition were confirmed by the DEMS measurements, which support the other measurements in this work, challenges with the use of FSI/DFOB anions were also encountered. We encountered specific problems with the electrolytes tested, as the electrolyte salts caused pitting corrosion of the steel cell housing on the cathode side (stainless steel 316Ti). This known problem is related to FSI containing salts,^[^ ^45^ ^]^ and has recently been studied for a NaFSI:NaPF_6_ mixed salt electrolyte,^[^ ^46^ ^]^ showing that stainless steel is more susceptible to the corrosion than the Al current collector. At the onset of the corrosion plateau in the measurements with 4.3 V cut‐off, the formation of SO_2_ was observed. This is more pronounced for the pure NaFSI electrolyte. As NaFSI is the only source of sulfur and NaFSI plays a major role for the steel corrosion the SO₂ is likely a product of the corrosion reaction. A more detailed discussion on the steel casing corrosion, the pseudo‐quantification of the gases, and other specific aspects observed for the electrolytes can be found in the supporting information (see Figure S12, Supporting Information).

## 3 Conclusion

In this work, we demonstrated that the simultaneous use of NaFSI and NaDFOB in PC represents an interesting and effective strategy for the realization of low‐fluorinated electrolytes for SIBs. The investigated electrolytes display good transport and thermal properties as well as large electrochemical stabilities. Furthermore, when the concentration of NaDFOB is high enough, they also display the ability to suppress the anodic dissolution of Al current collector. The use of these dual salt electrolytes in combination with P2‐Na_2/3_Al_1/9_Fe_1/9_Mn_2/3_Ni_1/9_O_2_ cathodes operating 4.3 V vs Na^+^/Na, results in high specific capacity and excellent cycling stability (capacity retention higher than 90% after 100 cycles). To the best of our knowledge, this performance is among the highest reported for this type of cathode material at 4.3 V vs Na^+^/Na.^[^ ^23^, ^36^, ^37^ ^]^ XPS and DEMS analysis indicated that this high performance is related to the formation of a thin, homogeneous and inorganic‐rich CEI. Considering these results, the development of low‐fluorinated electrolytes based on NaFSI and NaDFOB appears as a very promising strategy for the realization of high‐performance SIBs.

## 4 Experimental Section

### Cathode Material Synthesis and Electrode Preparation

P2‐ Na_2/3_Al_1/9_Fe_1/9_Mn_2/3_Ni_1/9_O_2_ (here on named as P2‐AFMNO), was synthesized in the labs following a three‐step synthesis route by i) precipitation of a spherical double layer hydroxide precursor with a Al: Fe: Mn: Ni ratio of 1:1:6:1 ii) dry mixing of that precursor with Na_2_CO_3_ to obtain a Na: M ratio of 2:3 (with M = Al, Fe, Mn, Ni) and iii) calcination of the mixture at 950 °C for 10 h in synthetic air (20 vol.% O_2_ in Ar). A detailed description of the synthesis route and characterization of cathode active material is provided in the Supporting Information. After calcination, the obtained cathode active material was directly transferred into a Büchi glass oven, where it was kept at 200 °C and dynamic vacuum (∼ 2 × 10^−2^ Pa) overnight. Subsequent powder handling and electrode preparation was performed in the same glovebox to avoid any contact with ambient air.

Cathode composite electrodes were prepared by dispersing the cathode active material, the conductive agent SuperP‐Li (Imerys Graphite & Carbon) and the binder polyvinylidene difluoride (PVDF, Solvay Solef P5130) in the mass ration of 93:3:4 in an appropriate amount of anhydrous N‐methyl‐2‐pyrrolidone (Sigma–Aldrich). The obtained slurry was casted on aluminum current collector foils using the doctor blade technique. The dried electrode sheets were roll‐pressed with a lab‐calender (Sumeet) at a speed of 1 m min^−1^, a line pressure of 10 N mm^−1^ and a temperature of 100 °C. From these electrode sheets, electrodes with a diameter of 12 mm were punched and subsequently dried in a Büchi glass oven at 80 °C and dynamic vacuum (1 × 10^−1^ Pa) overnight. Typical electrodes exhibit an active material mass loading of ≈23.2 mg cm^−2^. SEM images of electrodes as top‐view and electrode cross sections are available in Figures S15 and S16 of Supporting Information. The SEM images of electrodes after electrochemical testing can be found in Figures S17 and S18 (Supporting Information).

### Electrolyte Preparation

NaFSI (BLDpharm, 99.97%) and NaDFOB (Sigma Aldrich, ≥99%) salts were used as received. The solvent PC (Sigma–Aldrich, anhydrous, 99.7%) was dried prior to utilization over molecular sieves (3 Å) for 3 days. Electrolytes were prepared inside an argon filled glovebox (MBraun, H_2_O < 5 ppm, O_2_ < 5 ppm) by adding PC to the requisite amount of salt(s) and dissolving them using a magnetic stirrer. The maximum solubility of NaDFOB in propylene carbonate (PC) is 1 m.

### Chemical‐Physical Characterization

*Conductivity*: The ionic conductivity of the electrolytes was determined in a temperature range of −30 to 80 °C by measuring the alternating current resistance (Modulab XM ECS potentiostat) of a cell with two parallel platinum electrodes and a known cell constant as described in the literature.^[^ ^47^ ^]^

*Density*: Density values were measured from 0 to 80 °C by using a DMA 4100M density meter (Anton Paar), whereby 0.5 mL electrolyte sample was applied.

*Viscosity*: The viscosity was measured in the Modular Compact rheometer MCR 102 (Anton Paar RheoCompass) from −20 to 80 °C at a shear rate of 1000 s^−1^, whereby 0.35 mL electrolyte sample was applied. The measurements were carried out according to the procedure described in the literature.^[^ ^47^ ^]^

*Thermal Stability*: Thermogravimetric analysis (TGA) is used to investigate the thermal stability of electrolytes by using a PerkinElmer STA 6000. The device required a purge under nitrogen for one hour before starting the measurement. Electrolytes were tested and analyzed under nitrogen with a gas flow of 30 mL min^−1^ and a gas pressure of 2.5 bar. Thermal ramp measurements were carried out between 30 and 550 °C, and with a heating rate of 10 °C min^−1^. Isothermal measurement were performed at 60 °C for 24 h.

### Electrochemical Characterization

*Electrochemical stability window*: Three‐electrode Swagelok‐type cells were assembled inside an argon filled glovebox (MBraun, H_2_O < 5 ppm, O_2_ < 5 ppm) using a platinum metal disc as working electrode, a self‐standing oversized activated carbon disk as counter electrode and sodium metal as reference electrode. In this test, 150 µL of electrolyte and a glass fiber separator (Whatman GF/D) were used. Linear sweep voltammetry (LSV) at 1 mV s^−1^ (±0.1 mA cm^−2^ current threshold) was applied using a BioLogic VMP‐3 potentiostat to investigate the electrochemical stability window.

*Anodic dissolution*: The cell set‐up consisted in three‐electrode Swagelok‐type cells composed of an original aluminum foil as working electrode, sodium metal as counter electrode and reference electrode. In this test 300 µL of electrolyte and glass fiber separators (Whatman GF/D) were used. The cells were scanned for 10 cycles at 0.5 mV s^−1^ (2.3 to 4.3 V vs Na^+^/Na) and the potential was held for 3 h at 4.3 V of each cycle.

*Galvanostatic cycling*: The P2‐AFMNO electrodes and the three electrolytes were tested in a half‐cell configuration (three electrode Swagelok cells) by using sodium metal as counter and reference electrodes. Galvanostatic charge/discharge tests were performed between 2.3 and 4.3 V versus Na^+^/Na at C‐rates of 0.1 C, 0.2 C, 0.33 C, 0.5 C, 1 C (1 C: 75 mAh g^−1^), and five cycles at each current, where 2.3 V equals the OCV of the cell after assembly and soaking. Stability was performed at 0.5 C between 2.3–4.3 V versus Na^+^/Na. All electrochemical tests were performed at room temperature.

### Surface Characterization

The surface chemistry of Al current collectors at 4.3 V versus Na^+^/Na and cycled P2‐Na_2/3_Al_1/9_Fe_1/9_Mn_2/3_Ni_1/9_O_2_ cathode electrodes in the three electrolytes were investigated by means of X‐ray photoelectron spectroscopy (XPS). The measurements were conducted at SPECS XPS using a monochromatic Al Kα (hν = 1.487 eV) X‐ray source and a Phoibos 150 XPS spectrometer (Surface concept) equipped with a microchannel plate and Delay Line Detector (DLD). The Al 2p, F 1s, C 1s, S 2p, Na 1s, and B 1s high‐resolution scans were acquired in a fixed analyzer transmission mode with an X‐ray power source of 200 W (15 kV), a pass energy of 30 eV, and 0.1 eV energy steps. The XPS spectra were fitted using CasaXPS software, considering a nonlinear Shirley‐type background and 70% Gaussian and 30% Lorentzian line shape.^[^ ^38^ ^]^ Calibration of the binding energy was carried out using the C 1s adventitious carbon at 284.8 eV.^[^ ^38^ ^]^

### Differential Electrochemical Mass Spectrometry

The detailed description of the differential electrochemical mass spectrometry (DEMS) setup and the data evaluation was published elsewhere.^[^ ^48^ ^]^ For the DEMS measurements the same P2‐AFMNO cathodes, just with a different wet film thickness of 230 µm and different size of 11.3 cm^2^, as well as 1.5 mL of the same electrolytes where used. Whatman GF/A glass microfiber filters were used as separators, and sodium metal as reference and counter electrodes.

## Conflict of Interest

The authors declare no conflict of interest.

## Supporting information



Supporting Information

## Data Availability

The data that support the findings of this study are available from the corresponding author upon reasonable request.

## References

[1] a) M. D. Slater , D. Kim , E. Lee , C. S. Johnson , Adv Funct Mater. 2013, 23, 947;

[2] a) A. Eftekhari , D.‐W. Kim , J. Power Sources 2018, 395, 336;

[3] a) H. Rostami , J. Valio , P. Suominen , P. Tynjälä , U. Lassi , Chem. Eng. J. 2024, 495, 153471; 10.1002/cphc.202400459 PMC11648842 39264359

[4] S. Qiao , Q. Zhou , M. Ma , H. K. Liu , S. X. Dou , S. Chong , ACS Nano 2023, 17, 11220. 37289640 10.1021/acsnano.3c02892

[5] a) Y. Lu , L. Wang , J. Cheng , J. B. Goodenough , Chem. Commun. 2012, 48, 6544; 10.1039/c2cc31777j 22622269

[6] a) O. A. Drozhzhin , I. V. Tertov , A. M. Alekseeva , D. A. Aksyonov , K. J. Stevenson , A. M. Abakumov , E. V. Antipov , Chem. Mater. 2019, 31, 7463;

[7] a) J.‐J. Braconnier , C. Delmas , C. Fouassier , P. Hagenmuller , Mater. Res. Bull. 1980, 15, 1797;

[8] a) P. Barnes , K. Smith , R. Parrish , C. Jones , P. Skinner , E. Storch , Q. White , C. Deng , D. Karsann , M. L. Lau , J. J. Dumais , E. J. Dufek , H. Xiong , J. Power Sources 2020, 447, 227363;

[9] a) Y. Wang , Z. Wu , F. M. Azad , Y. Zhu , L. Wang , C. J. Hawker , A. K. Whittaker , M. Forsyth , C. Zhang , Nat. Rev. Mater. 2024, 9, 119;

[10] S. A. Solvionic , Sodium bis(fluorosulfonyl)imide Safety Data Sheet , https://solvionic.com/en/metallic‐salts/6061‐sodiumi‐bisfluorosulfonylimide.html.

[11] Sigma‐Aldrich, Sodium hexafluorophosphate Safety Data Sheet , https://www.sigmaaldrich.com/DE/de/product/aldrich/936022.

[12] H.‐B. Han , S.‐S. Zhou , D.‐J. Zhang , S.‐W. Feng , L.‐F. Li , K. Liu , W.‐F. Feng , J. Nie , H. Li , X.‐J. Huang , J. Power Sources 2011, 196, 3623.

[13] a) E. Kramer , S. Passerini , M. Winter , ECS Electrochem. Lett. 2012, 1, C9;

[14] J. Chen , Z. Huang , C. Wang , S. Porter , B. Wang , W. Lie , H. K. Liu , Chem. Commun. 2015, 51, 9809. 10.1039/c5cc02901e 25987231

[15] L. Gao , J. Chen , Y. Liu , Y. Yamauchi , Z. Huang , X. Kong , J. Mater. Chem. A 2018, 6, 12012.

[16] M. Hashimov , A. Hofmann , Batteries 2023, 9, 530.

[17] X. Liu , J. Zhao , H. Dong , L. Zhang , H. Zhang , Y. Gao , X. Zhou , L. Zhang , L. Li , Y. Liu , S. Chou , W. Lai , C. Zhang , S. Chou , Adv. Funct. Mater. 2024, 34, 2402310.

[18] M. He , L. Zhu , G. Ye , Y. An , X. Hong , Y. Ma , Z. Xiao , Y. Jia , Q. Pang , Angew. Chem. 2024, 136, 202401051. 10.1002/anie.202401051 38469954

[19] a) Z. Wang , J. Diao , G. Henkelman , C. B. Mullins , Adv. Funct. Mater. 2024, 34;

[20] H. M. Law , J. Yu , S. C. Kwok , G. Zhou , M. J. Robson , J. Wu , F. Ciucci , Energy Storage Mater. 2022, 46, 182.

[21] L. Huang , Q. Qiu , M. Yang , H. Li , J. Zhu , W. Zhang , S. Wang , L. Xia , P. Müller‐Buschbaum , ACS Appl. Mater. Interfaces 2024, 16, 46392. 39172040 10.1021/acsami.4c10970

[22] a) Y. Wang , Y. Wang , Y. Xing , C. Jiang , Y. Pang , H. Liu , F. Wu , H. Gao , J. Mater. Chem. A 2023, 11, 19955;

[23] W. Yin , Z. Huang , T. Zhang , T. Yang , H. Ji , Y. Zhou , S. Shi , Y. Zhang , Energy Storage Mater. 2024, 69, 103424.

[24] a) C. Geng , D. Buchholz , G.‐T. Kim , D. V. Carvalho , H. Zhang , L. G. Chagas , S. Passerini , Small Methods 2019, 3, 1800208;

[25] a) L. G. Chagas , S. Jeong , I. Hasa , S. Passerini , ACS Appl. Mater. Interfaces 2019, 11, 22278; 31144802 10.1021/acsami.9b03813

[26] G. G. Eshetu , S. Grugeon , H. Kim , S. Jeong , L. Wu , G. Gachot , S. Laruelle , M. Armand , S. Passerini , ChemSusChem 2016, 9, 462. 26834069 10.1002/cssc.201501605

[27] a) I. Cekic‐Laskovic , N. von Aspern , L. Imholt , S. Kaymaksiz , K. Oldiges , B. R. Rad , M. Winter , Top. Curr. Chem. 2017, 375, 37; 10.1007/s41061-017-0125-8 28299728

[28] a) S. Huang , S. Wang , G. Hu , L.‐Z. Cheong , C. Shen , Appl. Surf. Sci. 2018, 441, 265;

[29] K. Wang , S. Gao , L. Li , L. Wang , X. Yang , X. Li , W. Lü , Chemistry 2024, 30, 202400803. 10.1002/chem.202400803 38752562

[30] K. S. Teoh , M. Melchiorre , S. Darlami Magar , M. Hermesdorf , D. Leistenschneider , M. Oschatz , F. Ruffo , J. L. Gómez Urbano , A. Balducci , Adv. Mater. 2024, 36, 2310056. 10.1002/adma.202310056 38252812

[31] J. Lee , Y. Lee , J. Lee , S.‐M. Lee , J.‐H. Choi , H. Kim , M.‐S. Kwon , K. Kang , K. T. Lee , N.‐S. Choi , ACS Appl. Mater. Interfaces 2017, 9, 3723. 28067499 10.1021/acsami.6b14878

[32] L. O. S. Colbin , C. A. Hall , A. S. Etman , A. Buckel , L. Nyholm , R. Younesi , Energy Adv. 2024, 3, 143.

[33] E. Hoque , J. A. DeRose , G. Kulik , P. Hoffmann , H. J. Mathieu , B. Bhushan , J. Phys. Chem. B 2006, 110, 10855. 16771337 10.1021/jp061327a

[34] J. Han , A. Mariani , H. Zhang , M. Zarrabeitia , X. Gao , D. V. Carvalho , A. Varzi , S. Passerini , Energy Storage Mater. 2020, 30, 196.

[35] D. C. Messina , B. S. Eller , P. A. Scowen , R. J. Nemanich , J. Vacuum Sci. Technol. A 2022, 40, 012404.

[36] B. Sambandam , M. H. Alfaruqi , S. Park , S. Lee , S. Kim , J. Lee , V. Mathew , J.‐Y. Hwang , J. Kim , ACS Appl. Mater. Interfaces 2021, 13, 53877. 34743513 10.1021/acsami.1c15394

[37] X.‐L. Li , T. Wang , Y. Yuan , X.‐Y. Yue , Q.‐C. Wang , J.‐Y. Wang , J. Zhong , R.‐Q. Lin , Y. Yao , X.‐J. Wu , X.‐Q. Yu , Z.‐W. Fu , Y.‐Y. Xia , X.‐Q. Yang , T. Liu , K. Amine , Z. Shadike , Y.‐N. Zhou , J. Lu , Adv. Mater. 2021, 33, 2008194. 10.1002/adma.202008194 33645858

[38] M. C. Biesinger , Appl. Surf. Sci. 2022, 597, 153681.

[39] G. Beamson , D. Briggs , High resolution XPS of organic polymers. The Scienta ESCA300 database, Wiley, Chichester England, New York 1992.

[40] C. D. Wagner , A. V. Naumkin , A. Kraut‐Vass , J. W. Allison , C. J. Powell , J. R. Rumble , NIST Standard Reference Database 20, Version 3.4 (web version), National Institute of Standards and Technology, 2003.

[41] M. Zarrabeitia , L. Gomes Chagas , M. Kuenzel , E. Gonzalo , T. Rojo , S. Passerini , M. Á. Muñoz‐Márquez , ACS Appl. Mater. Interfaces 2019, 11, 28885. 31318528 10.1021/acsami.9b07963

[42] a) M. A. Muñoz‐Márquez , M. Zarrabeitia , E. Castillo‐Martínez , A. Eguía‐Barrio , T. Rojo , M. Casas‐Cabanas , ACS Appl. Mater. Interfaces 2015, 7, 7801; 25811538 10.1021/acsami.5b01375

[43] X. Hu , Y. Wang , Y. Qiu , X. Yu , Q. Shi , Y. Liu , W. Feng , Y. Zhao , Chem., Asian J. 2024, 19, 202300960. 10.1002/asia.202300960 38143238

[44] S. Tanuma , C. J. Powell , D. R. Penn , Surf. Interface Analys. 2011, 43, 689.

[45] T. Evans , J. Olson , V. Bhat , S.‐H. Lee , J. Power Sources 2014, 269, 616.

[46] W. Fan , W. Wang , Q. Xie , X. He , H. Li , J. Zhao , J. Nan , Chemistry 2024, 30, 202401321. 10.1002/chem.202401321 38801410

[47] L. H. Heß , A. Balducci , ChemSusChem 2018, 11, 1919. 29729088 10.1002/cssc.201800375

[48] a) J. Geisler , Online gas analysis of electrochemical reactions, Humboldt‐Universität zu Berlin, Berlin, Germany 2023;

