# Supplementary material for: The Impact of Dual‐Salt Electrolyte with Low Fluorine Content on the Performance of Layered Transition Metal Oxides for Sodium‐Ion Batteries
Source: Small. 2025 May 8;22(12):2410704. doi: 10.1002/smll.202410704 (PMC12934380; doi:10.1002/smll.202410704)
Supplement: Supplementary file 1 — Supporting Information [file SMLL-22-2410704-s001.docx]

**Supplementary information**
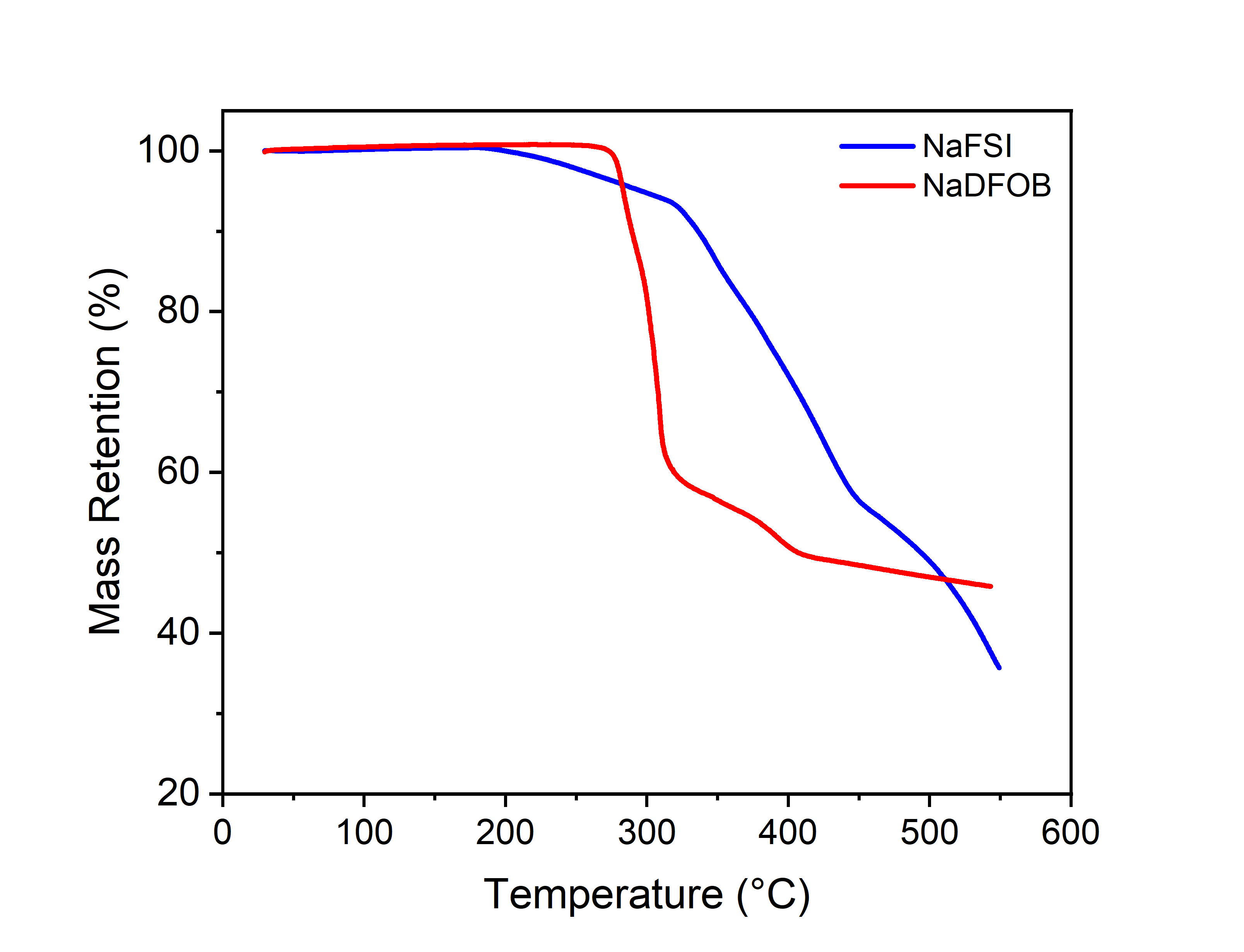


**Figure S1** TGA test of Pure NaFSI and NaDFOB



 **Figure S2** The isothermal measurement of the three electrolytes at 60 °C





**Figure S3** Anodic dissolution of 3 electrolytes

**
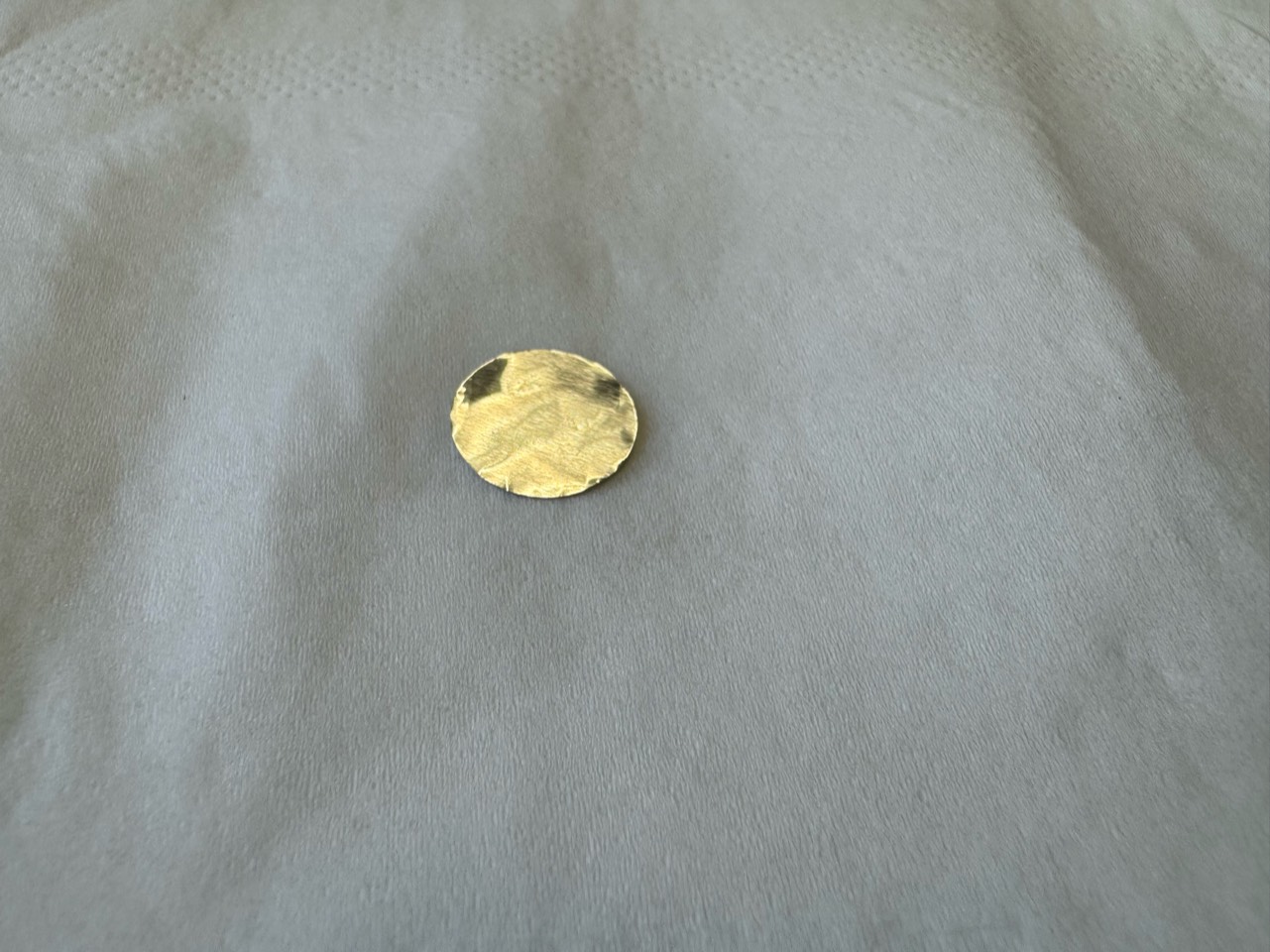
**

**Figure S4** The Original Al foil


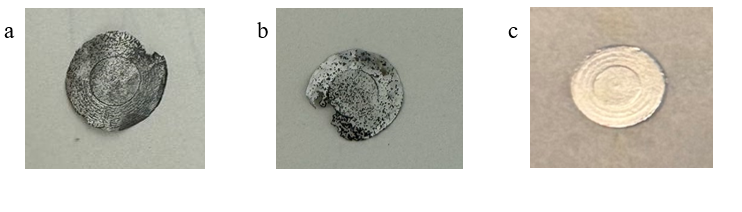


d f e


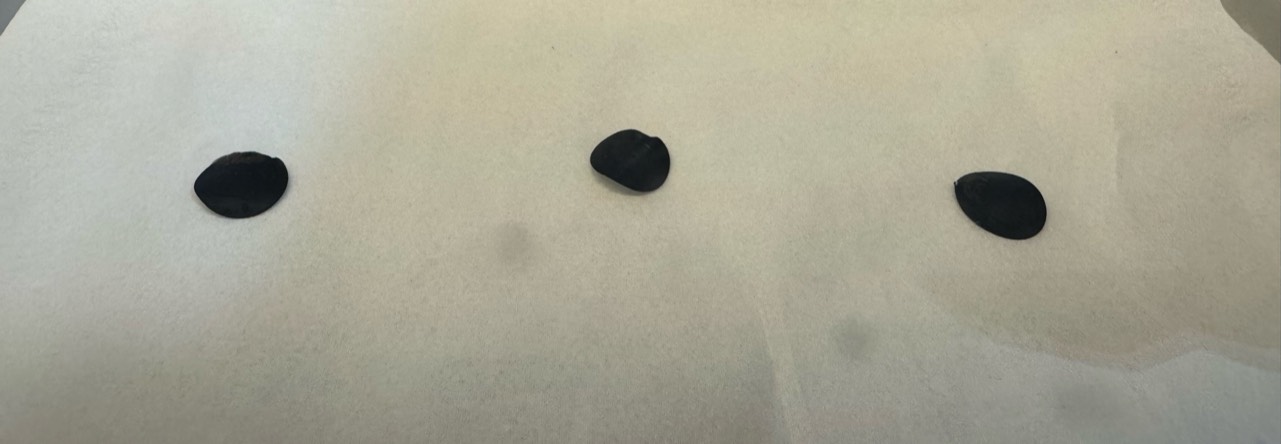

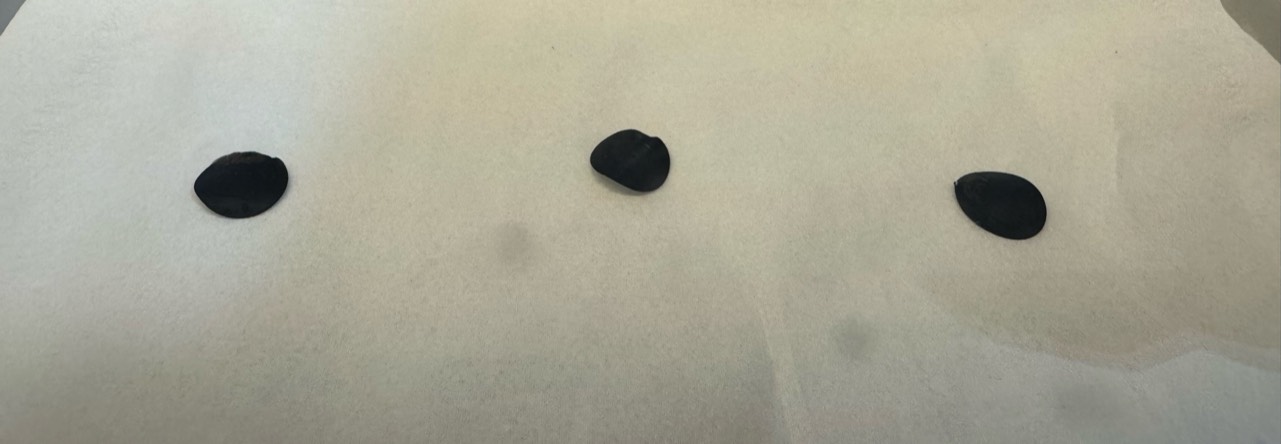

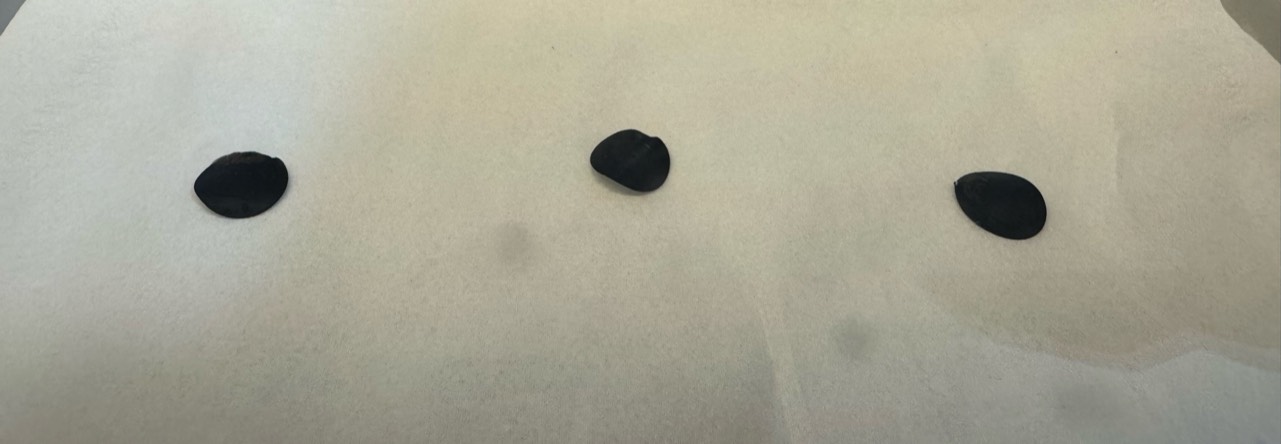


h i j


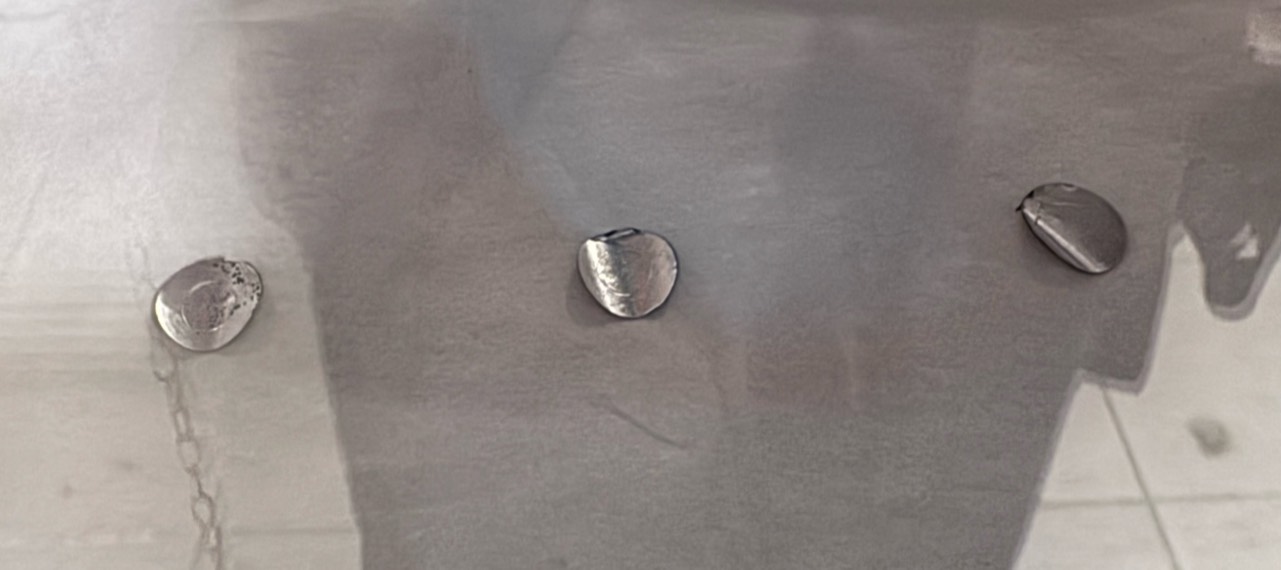
 **
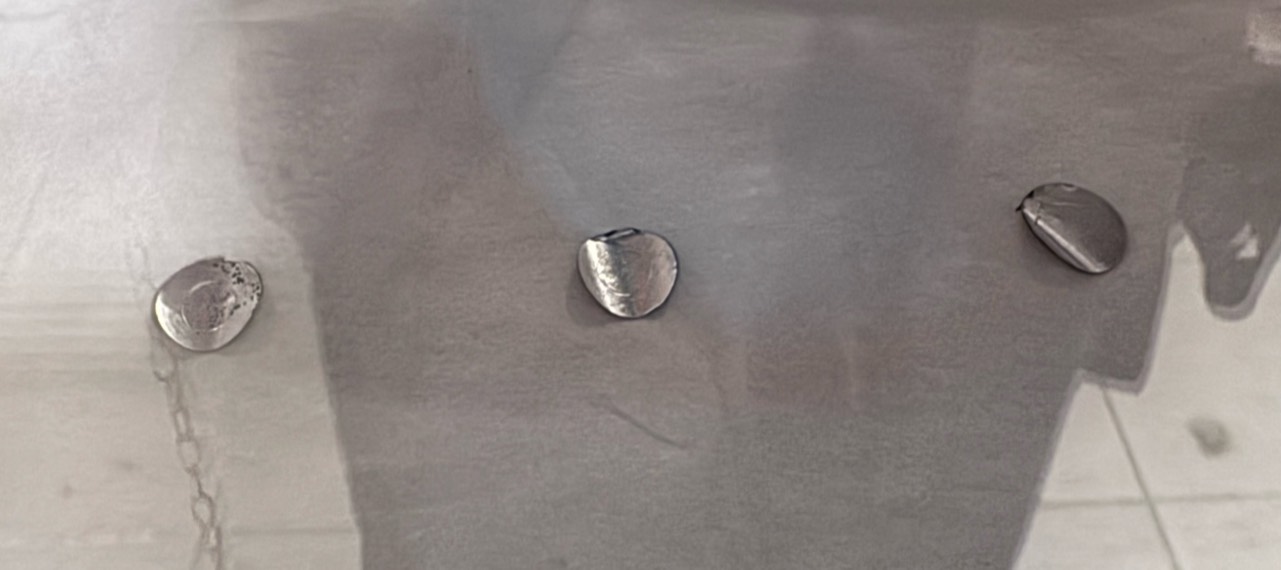
**
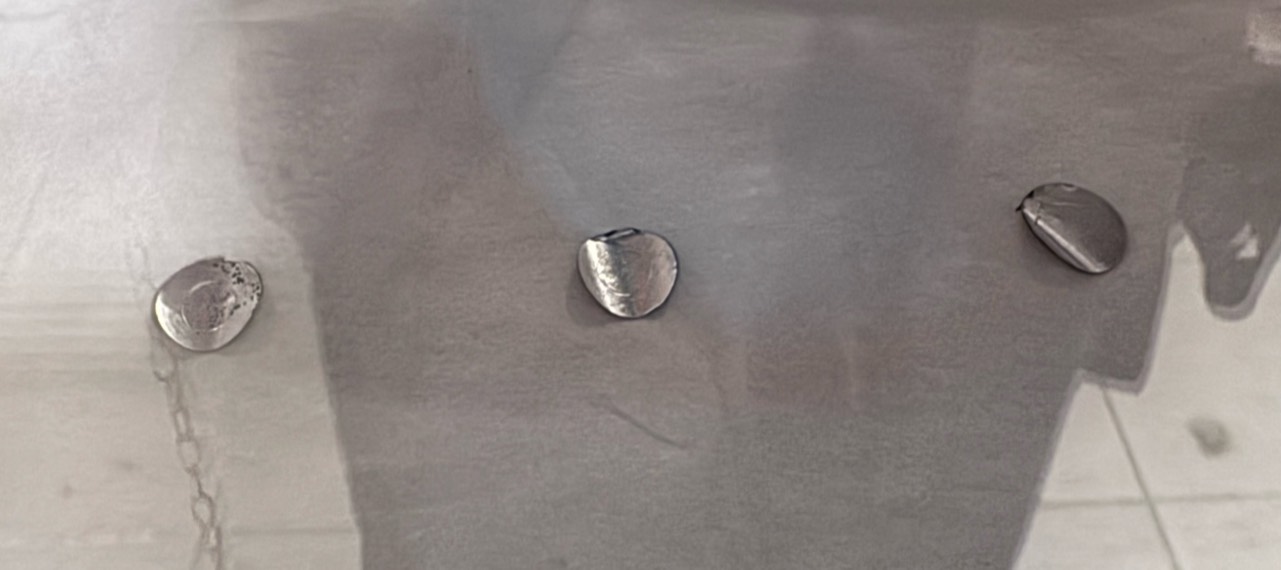


**Figure S5** The Al foil after anodic dissolution test a. 1 M NaFSI in PC; b. 0.8 M NaFSI + 0.2 M NaDFOB in PC; c. 0.5 M NaFSI + 0.5 M NaDFOB in PC and the cathode material after cycling d. 1 M NaFSI in PC; e. 0.8 M NaFSI + 0.2 M NaDFOB in PC; f. 0.5 M NaFSI + 0.5 M NaDFOB and The Al foil side of cathode material after cycling h. 1 M NaFSI in PC; i. 0.8 M NaFSI + 0.2 M NaDFOB in PC; j. 0.5 M NaFSI + 0.5 M NaDFOB in PC





**Figure S6** The C-rate test for a. 1 M NaFSI in PC; b. 0.8 M NaFSI + 0.2 M NaDFOB in PC; c. 0.5 M NaFSI + 0.5 M NaDFOB in PC





**Figure S7** The C-rate test for a. 1 M NaFSI in PC; b. 0.8 M NaFSI + 0.2 M NaDFOB in PC; c. 0.5 M NaFSI + 0.5 M NaDFOB in PC





**Figure S8**a**.** The evolution of the differential capacity of the electrodes in first cycle; b. the evolution of the differential capacity of the electrodes after 100 cycles.







**Figure S9**. Long cycling term performance of 0.8M NaFSI+0.2M NaDFOB in PC and 0.5M NaFSI+0.5M NaDFOB in PC


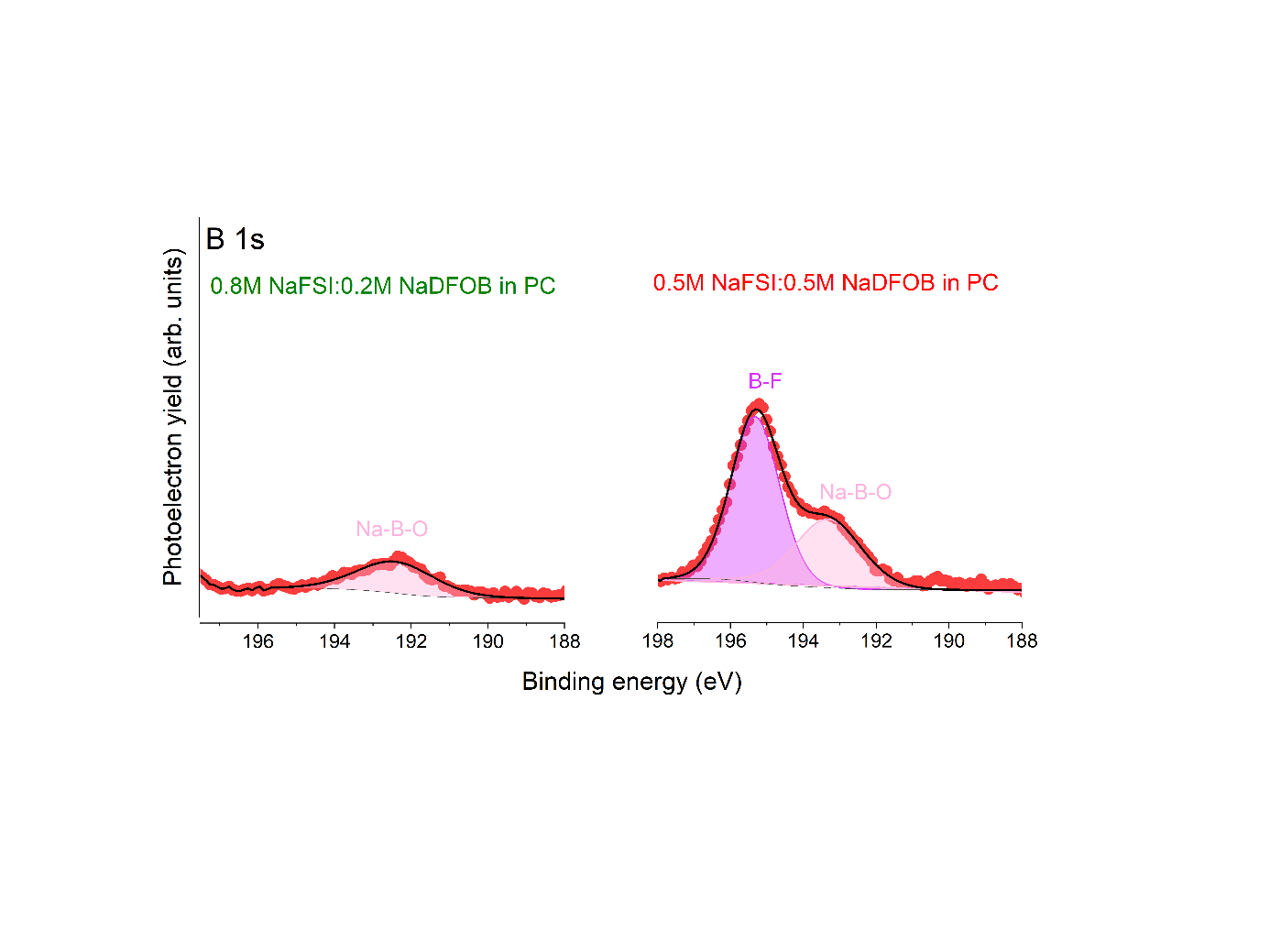


**Figure S10**. B 1s region (XPS) of P2-AFMNO electrode tested in 0.8 M NaFSI + 0.2 M NaDFOB in PC and 0.5 M NaFSI + 0.5 M NaDFOB in PC electrolytes.


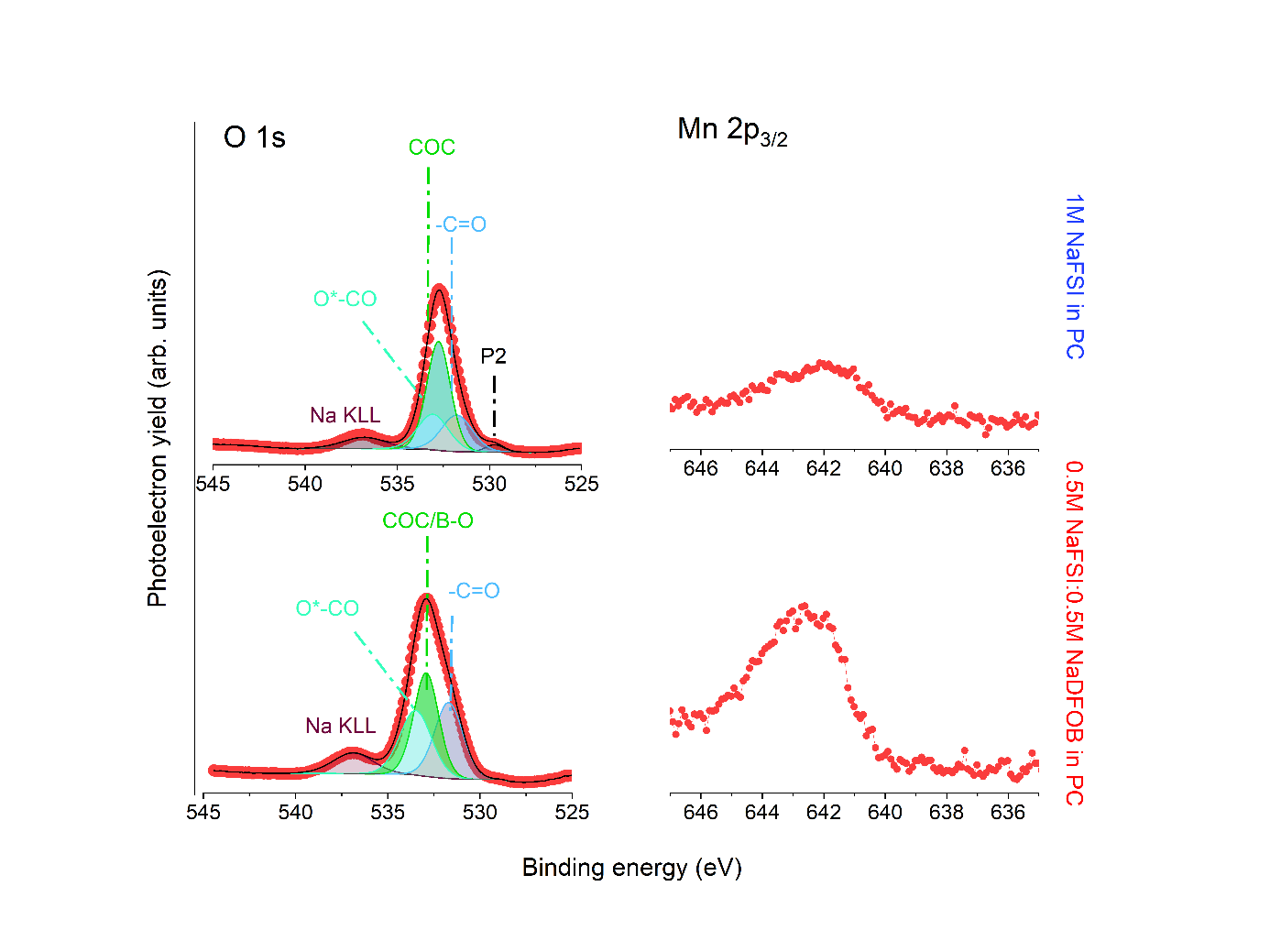


**Figure S11**. O 1s and Mn 2p photoelectron regions of P2-AFMNO electrode tested in 1 M NaFSI in PC and 0.5 M NaFSI + 0.5 M NaDFOB in PC electrolytes.

**Supplementary Information: DEMS measurements**

The main limitation that was found is that the DEMS operando cell material was not compatible with the NaFSI electrolyte. As a result of the steel corrosion a brownish-black discoloration of the electrolyte and dark deposits on the sodium counter metal electrode were observed post-mortem. Due to the corrosion on the cell casing, it is difficult to get a clear picture of the side reactions occurring due to the active material and the aluminum current collector. It is the aim of further studies, to get the parts of the operando DEMS cell machined form a different material, which might be more resistant to the corrosion with NaFSI, it was not feasible for this work.

Due to the corrosion, it was not possible to reach the upper cut-off potential of 4.3 V vs. Na^+^/Na for both electrolytes. Therefore, the measurements were repeated with an upper cut-off of 3.9 V. Under these conditions a stable cycling of the 0.5 M NaFSI + 0.5M NaDFOB electrolyte was possible, while the pure NaFSI electrolyte failed during the third charge, due to corrosion, which is an indication that the NaDFOB also protects the steel from corroding.

In addition to corrosion, a background drift was observed with these electrolytes, affecting the signals for m/z values 1 to 5. These are affected by the background signal from the helium carrier gas. As a result, the background for these signals could not be fully subtracted, leading to difficulties in proper quantification of the H_2_ signal. Therefore, the H_2_ signals must be considered pseudo-quantitative. The amounts of SO_2_ and NO are also pseudo-quantitative, as the system was not calibrated with calibration gas mixtures of these gases. Instead, an arbitrary value of 1.5E-2 was chosen, based on the fact, that all properly calibrated gases have values in the range of 9.5E‑3 – 2.5E‑2 for the method used.

A strong evolution of NO was observed in the 4.3 V NaFSI: NaDFOB measurement. If the pseudo-quantification is appropriate, this would be the strongest gas formation of all the measurements. The onset of the NO evolution is around 3.3 V, where an additional plateau in the voltage profile is observable only in this measurement. None of the other measurements showed detectable NO formation. We hypothesis that this is a product of the reaction between the electrolyte and the steel surface, this could be a reason why this signal only appears in this measurement, as the responsible species could be depleted from the steel afterwards.


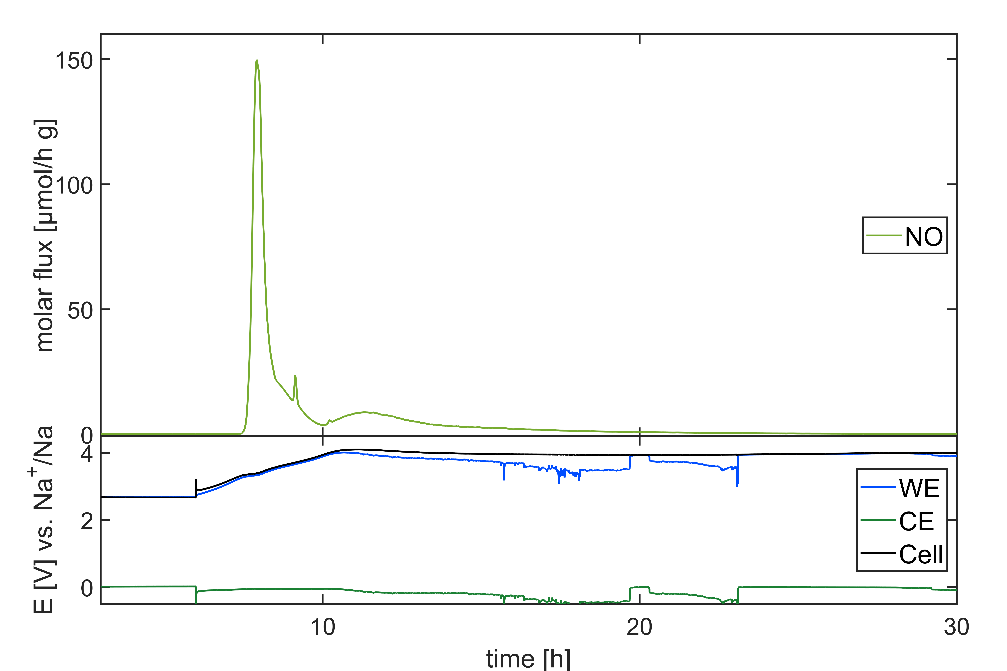


**Figure S12**. NO evolution in 0.5 M NaFSI + 0.5 M NaDFOB in PC with upper cut-off at 4.3 V. Potentials given for P2-AFMNO working electrodes (WE) and sodium metal counter electrode (CE) against sodium metal reference and the resulting cell potential (Cell). Although the total amounts of gas evolution are given for guidance, some measurements are only pseudo-quantitative.

**Cathode material synthesis**

The layered double hydroxide (or hydrotalcite-type) precursor [Al_1/9_Fe_1/9_Mn_2/3_Ni_1/9_(OH)_2_]^+2/9^[(NO_3_^-^)_2/9_ ∙ z H_2_O]^-2/9^ was synthesized by continuously feeding water, a deaerated solution of NaOH (Carl Roth), and a stoichiometric deaerated solution of Al(NO_3_)_3_ x 9 H_2_O, Fe(NO_3_)_3_ x 9 H_2_O, Mn(NO_3_)_2_ x 4 H_2_O and Ni(NO_3_)_2_ x 6 H_2_O (all Carl Roth) into the vigorously stirred tank reactor (CSTR, Volume 1 liter). Citric acid added to the metal solution served as chelating agent. ^[1]^ After the run-in period, the dispersion at the reactor outlet was collected and continuously washed and filtered to remove any residual salt solution. The dried precipitate was heated in a muffle furnace (Nabertherm) at 900 °C for 1 h in air to remove any crystal water from the hydrotalcite-type precursor transforming the precursor to an oxide. Subsequently, the precursor was dry mixed with a respective amount of Na_2_CO_3_ (Sigma Aldrich) and then heated in a box furnance (Carbolite Gero) at 950 °C for 10 h in synthetic air (20 vol.% O_2_ in Ar). After natural cooling to 200 °C, the obtained powder was directly transferred into a Büchi glass oven, where it was kept at 200°C and dynamic vacuum (~ 2 x 10^-2^ Pa) overnight. The obtained cathode active material was then relocated without any further contact with ambient air into an Ar-filled glovebox (MBraun, O_2_ < 0.1 ppm, H_2_O < 0.1 ppm), where subsequent powder handling and electrode preparation was performed.

**Characterization of cathode active material**

Elemental analysis of the obtained cathode active material was performed using inductively coupled plasma optical emission spectroscopy (ICP-OES, Spectro Arcos SOP) with a diluted *aqua regia* solution. The obtained chemical composition represented as a chemical formula for layered sodium transition metal oxides (Na_x_MO_2_ with M = Al, Fe, Mn, Ni) is Na_0.659_Al_0.115_Fe_0.113_Mn_0.660_Ni_0.113_O_2_, which is in good accordance with the targeted stoichiometry of Na_2/3_Al_1/9_Fe_1/9_Mn_2/3_Ni_1/9_O_2_. The crystal structure of the cathode active material was characterized using X-ray powder diffraction in Bragg-Brentano geometry on a Bruker D8Advance with a Cu X-ray source and a LynxEye XE-T detector. The obtained diffraction pattern is presented in Figure S11. The obtained diffraction pattern is similar to PDF 00-054-0839 and is well indexed with the hexagonal space group *P*6_3_/*mmc* (SG 194), indicating the phase pure P2-type^[2]^ crystalline structure. The morphology of the cathode active material was depicted using scanning electron microscopy on a Zeiss Leo 1530VP equipped with an Everhart-Thornley SE detector at 5 kV acceleration voltage as presented in Figure S15. The cathode active material consists of dense, round secondary particles in the range of 2 µm to 15 µm.


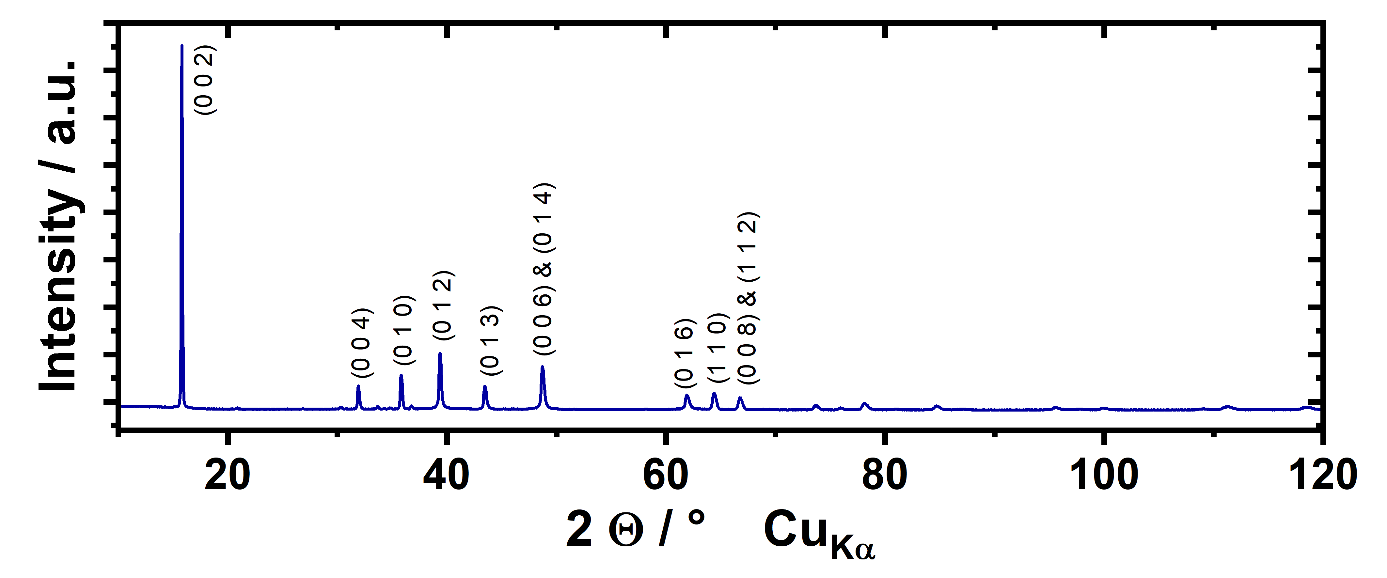
**Figure S13** Powder X-ray diffraction pattern of the cathode active material.


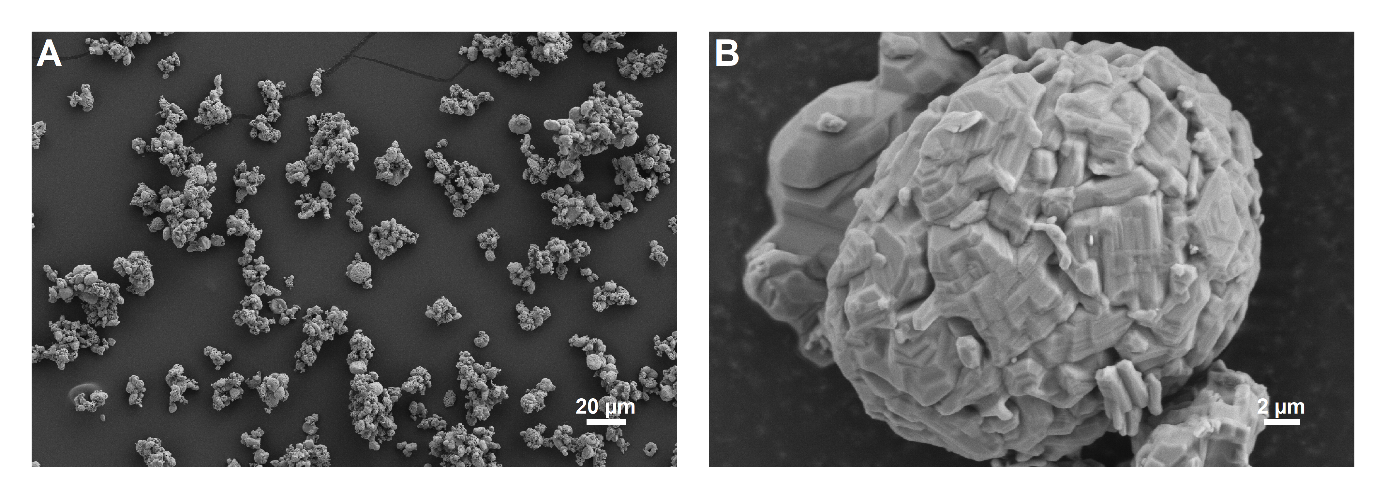
**Figure S14** SEM images of the as-prepared cathode active material.


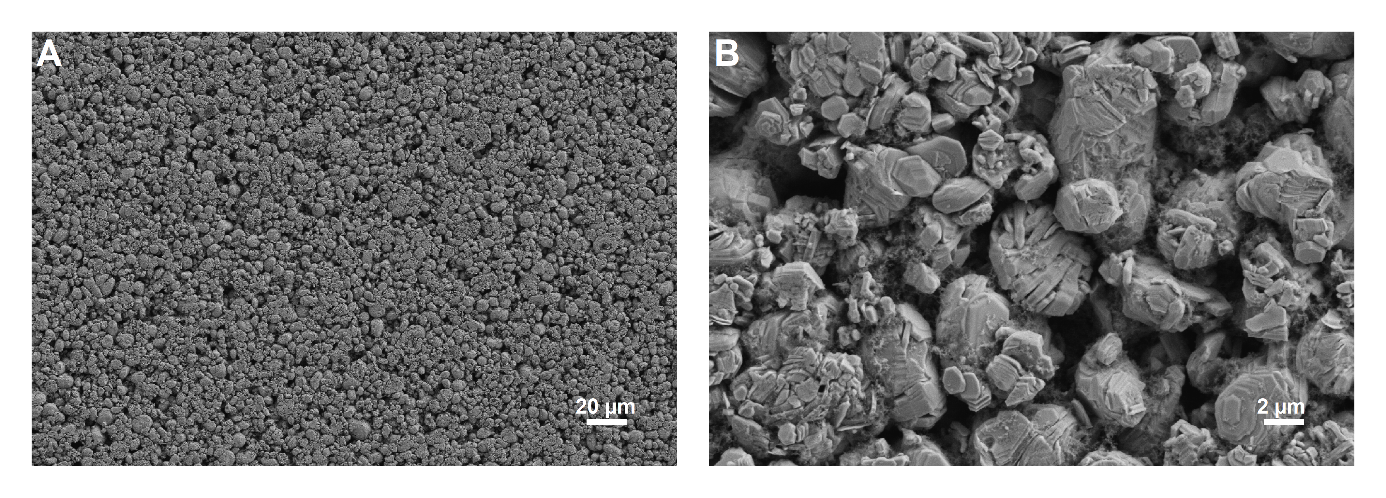


**Figure S15** SEM images of the cathode electrode as a top-view.

**
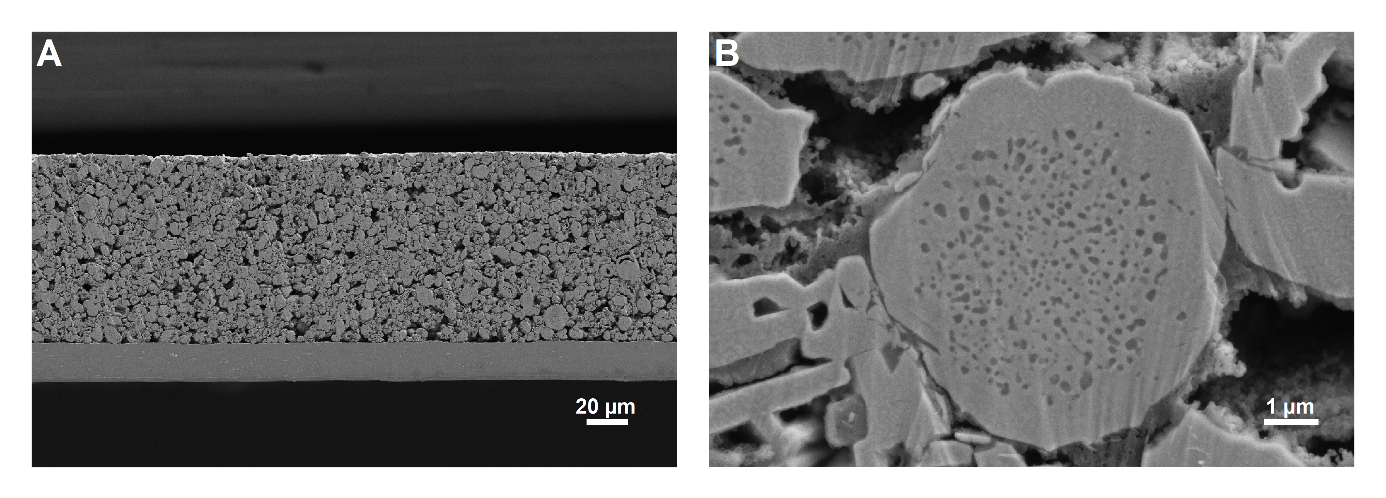
 Figure S16** SEM images of the cathode electrode as cross-section.

**
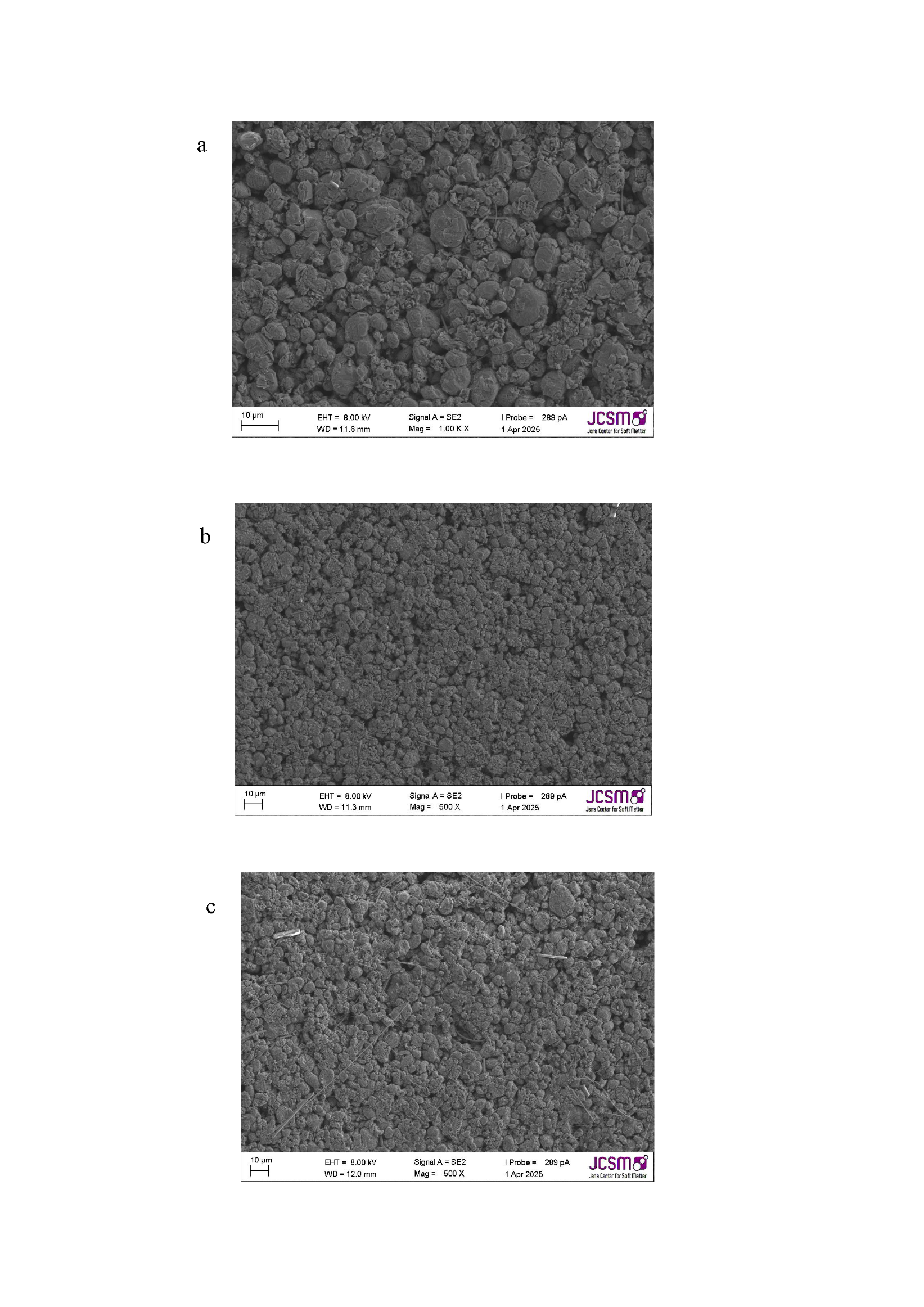
**

**Figure S17** SEM images of the cathode electrode after long-term test as a top-view: a. 1M NaFSI in PC; b. 0.8 M NaFSI + 0.2 M NaDFOB in PC; c. 0.5 M NaFSI + 0.5 M NaDFOB in PC


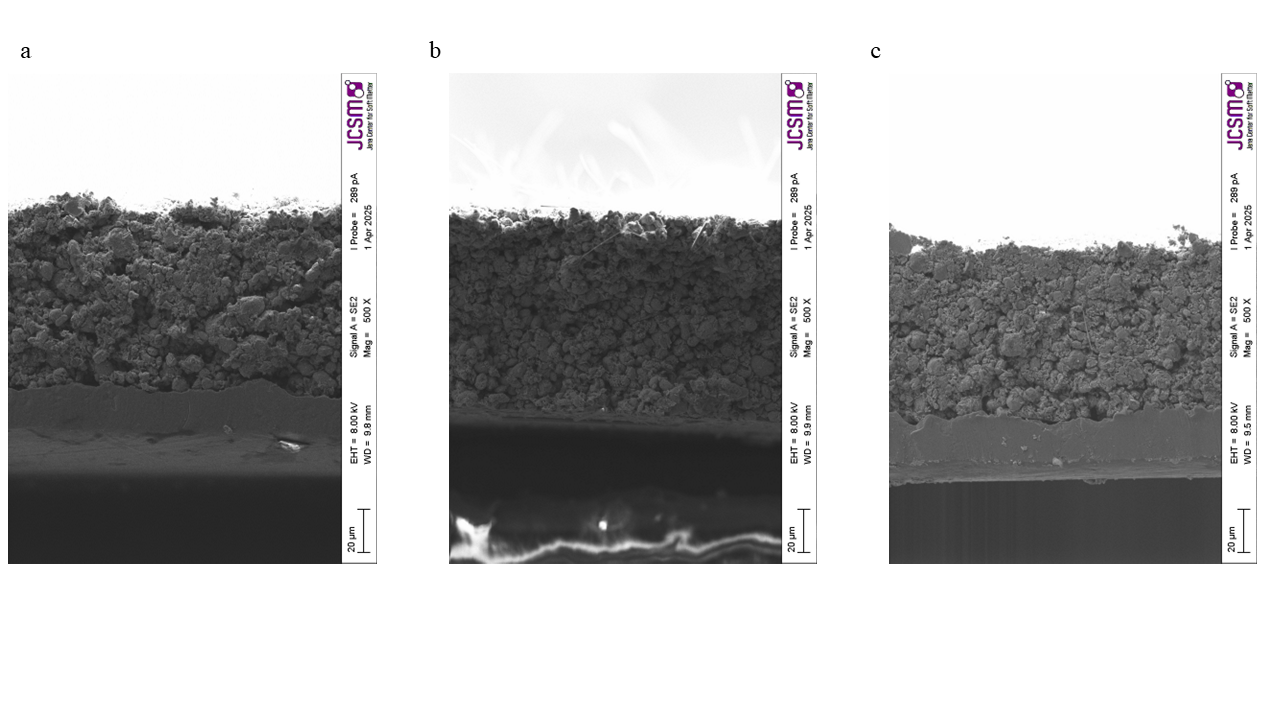
**Figure S18** SEM images of the cathode electrode after long-term test as as cross-section: a. 1M NaFSI in PC; b. 0.8 M NaFSI + 0.2 M NaDFOB in PC; c. 0.5 M NaFSI + 0.5 M NaDFOB in PC

**References**

[1] J. Lamb, A. Manthiram, *Chem. Mater.* **2020**, *32*, 8431.

[2] C. Delmas, C. Fouassier, P. Hagenmuller, *Physica B+C* **1980**, *99*, 81.
